# Timed Picture Naming Norms for Mandarin Chinese

**DOI:** 10.1371/journal.pone.0016505

**Published:** 2011-01-26

**Authors:** Youyi Liu, Meiling Hao, Ping Li, Hua Shu

**Affiliations:** 1 State Key Laboratory of Cognitive Neuroscience and Learning, Beijing Normal University, Beijing, China; 2 Center for Studies of Chinese as a Second Language, Beijing Language and Culture University, Beijing, China; 3 Department of Psychology, Pennsylvania State University, University Park, Pennsylvania, United States of America; University of Oxford, United Kingdom

## Abstract

The present study reports timed norms for 435 object pictures in Mandarin Chinese. These data include naming latency, name agreement, concept agreement, word length, and age of acquisition (AoA) based on children's naming and adult ratings, and several other adult ratings of concept familiarity, subjective word frequency, image agreement, image variability, and visual complexity. Furthermore, we examined factors that influence the naming latencies of the pictures. The results show that concept familiarity, AoA, concept agreement, name agreement, and image agreement are significant predictors of naming latencies, whereas subjective word frequency is not a reliable determinant. These results are discussed in light of picture naming data in other languages. An item-based index for the norms is provided in the Table S1.

## Introduction

Picture naming is a widely used paradigm in psycholinguistic research [1], and recently it has also become an important method in brain imaging studies [2], [3]. Normed picture naming data provide standardized tools that allow for the comparison of different studies addressing different theoretical questions. In this study, we report a first comprehensive normed dataset in Mandarin Chinese from the naming of 435 object line drawings. The dataset includes eleven variables: naming latency, name agreement (both in percentage and in H-statistics), adult rated age of acquisition (AoA), AoA based on children's speech, subjective word frequency, concept agreement, concept familiarity, image agreement, image variability, visual complexity, and word length.

The variables that influence picture naming have been extensively investigated in many languages since the 1980s (see [4], for a review). Some variables such as AoA and name agreement are found to determine picture-naming speed universally across languages [4], [5], [6]. Other variables are found to be more language specific and influence naming latencies differently in different languages. For example, Bates et al. [6] found that word length was a significant predictor of naming latencies in English, Bulgarian and Hungarian, but not in German, Spanish, and Italian. Thus, it is important to carry out norming studies to identify which variables influence which languages. Weekes, Shu, Hao, Liu and Tan [7] investigated the possible variables affecting picture naming in Mandarin Chinese used in Beijing and found that name agreement, concept familiarity, and adult rated AoA had significant contributions to naming latency. However, their findings were based on 144 pictures. In addition, the indices for majority of the variables might be outdated, since they were taken from Shu, Cheng and Zhang [8] collected twenty years ago. Bates et al. [6] reported the significance of name agreement and word frequency on the naming of 520 object names in Mandarin Chinese used in Taiwan, but they did not consider the full range of variables, especially adult rated AoA. The current study aims at providing a more comprehensive index of variables for picture naming by adding more potential variables (e.g., objective AoA based on child speech), and further examining the impacts of these variables on picture naming with 435 pictures.

AoA has been shown to be consistently significant in every published study on picture naming (see review in [4]). It is worth noting that the AoA measure used in most studies has been based on adult estimates of when different words are learned, since adult ratings are much easier to collect than objective AoA data based on children's picture naming performance as originally used by Morrison, Chappell and Ellis [9]. The validity of rated AoA has been confirmed in several studies. For example, Carroll and White [10] reported a correlation of 0.85 between rated AoA and a normative study of when children are able to read words; Gilhooly and Gilhooly [11] found a correlation of 0.93 between rated AoA and the rank order of words in the norms from the Mill Hill standardized vocabulary scale [12]. Furthermore, Morrison et al. [9] found a correlation of around 0.8 between children's naming performance and adult AoA ratings for 297 object pictures. Despite the consistency shown in these studies between adult rated AoA and objective AoA based on child data, recent studies, however, have also found that the objective AoA is a more powerful determinant of naming latency than rated AoA [13], probably because the former is less contaminated by other variables [14], [15]. One goal of the present study is to collect both types of AoA and analyze their relationships with other variables so that we can identify their predictive power for adult picture naming latency.

The question of whether word frequency contributes significantly to picture naming latency over and above AoA has also been very controversial in recent years (see [4]). In contrast to AoA, nearly 50% of the studies in the literature did not observe a significant effect of word frequency. For example, Weekes et al. [7] showed no frequency effect in the naming of 144 pictures. The lack of a significant frequency effect has often been attributed to the lack of statistical power due to the small number of picture items used in studies (see [16], [17], [18]). Other possible reasons have also been identified, such as the existence of different kinds of frequency measures that could affect experimental results. Barry, Morrison and Ellis [19] found that spoken and written frequency had similar significant effects on the naming of 195 pictures from the Snodgrass and Vanderwart [20] norms. Instead of objective word frequency (written or spoken), Lachman, Shaffer and Hennrikus [21] used subjective word frequency and observed both AoA and frequency effects in picture naming task. Subjective word frequency based on participants' own ratings has also been used as a proxy for word frequency [9], [22], [23]. This method usually requires participants to judge how frequently they encounter a word (in reading or in spoken language) on a Likert-like scale. The correlation between subjective word frequency and objective word frequency (written or spoken) has been high, though not perfect [22], [23]. The current study will use subjective rather than actual word frequencies to study picture naming, because many picture names cannot be easily found in existing Chinese word frequency dictionaries (which could be due to text sampling problems associated with frequency dictionaries; see [24]).

As in previous studies of picture naming, other potentially important variables such as concept familiarity, image agreement, image variability, and visual complexity were also included in our study. Concept familiarity refers to the familiarity of the concept depicted by the picture. Image agreement refers to the degree of similarity between the mental image generated by a participant to a given picture's name and the actual picture displayed. Image variability refers to the number of different images evoked by the name of a particular object. Visual complexity refers to the number of lines and details in the drawing. In what follows we first report the procedure with which we collected the indices of all the variables for 435 line-drawing pictures, and then analyze their relationships and their contributions to picture naming latencies with multiple regression analyses.

## Methods

### Ethics Statement

This study was reviewed and approved by the committee for the protection of subjects at Beijing Normal University. Written consent was also obtained from every adult participant or from parents of every child participant before the experiment according to established guidelines of the committee.

### Participants

#### Adults

273 native Chinese speakers (185 females) with a mean age of 22.5 years (range = 18–26 yrs) participated in name writing and several rating tasks. A separate group of 35 speakers (25 females) with a mean age of 21.4 years (range = 18–25 yrs) participated in the picture naming task. All participants were undergraduate or graduate students from Beijing Normal University. They had normal or corrected-to-normal vision, and reported no cognitive or motor problems. They were paid for their participation.

#### Children

442 children from two preschools and two elementary schools at Haidian District, Beijing (age range = 2.4 to 11 yrs) were asked to name aloud subgroups of 435 pictures (see Table 1 for details). They were rewarded with small toys after the experiment. All of them had normal or corrected-to-normal vision, and reported no attention or motor problems.

**Table 1:** Information about the children whose speech formed the basis of the objective AoA data.

	Before K.	K1	K2	K3	G1	G2	G3
Mean age (*yrs.*)	2.94	3.84	4.81	5.84	7.25	8.00	9.27
Min. (*yrs.*)	2.40	3.24	4.31	4.98	6.79	7.34	8.39
Max. (*yrs.*)	3.34	4.81	5.41	6.63	7.72	9.00	10.88
Number (*person*)	50	99	64	99	18	55	57

Note: *Before K.* – preschoolers before entering kindergarten; K1 – kindergarten level 1; K2 – kindergarten level 2; K3 – kindergarten level 3; G1 – elementary school grade 1; G2 – elementary school grade 2; G3 – elementary school grade 3.

### Materials

The 435 object line-drawings of the present study included 266 from Cycowicz, Friedman, Rothstein, and Snodgrass [25], 40 from Bonin, Peereman, Malardier, Meot, and Chalard et al. [26], 14 from Philadelphia Naming Test [27], 41 from Zhang and Yang [28] and 74 from other sources. The 266 pictures from Cycowicz et al. [25] are available at the website http://www.nyspi.cpmc.columbia.edu/nyspi/respaprs/picnorm.htm, and the other 169 pictures are available at PLoS One's Online Archive (see Files S1), or upon request from the authors. This collection of pictures from several sources enabled a large number of pictures to be involved and we made efforts to ensure that the pictures were familiar to Chinese speakers. The style of all 435 line drawings was similar to those in Snodgrass and Vanderwart [20]. Among the 435 pictures used here, 218 are identical or similar to the object pictures used in the International Picture Naming Project (IPNP; [29]), sometimes with only slight differences such as different angles of view.

### Procedures

First, 40 undergraduates were given the pictures printed on paper and instructed to write down the name of the picture that first came to their mind. If they could not provide the name of the picture, they were asked to answer if their inability to name was due to “Don't know the object (DKO)”, “Don't know the name of the object (DKN)”, or “Tip of the tongue (TOT)”. The dominant name, name agreement and concept agreement could be assigned or calculated for each picture based on the results of this task.

Second, another group of 48 undergraduates rated AoA for the dominant names of these pictures on a 7-point scale (see [30]), where 1 = 0–2 yrs, 2 = 3–4 yrs, 3 = 5–6 yrs, 4 = 7–8 yrs, 5 = 9–10 yrs, 6 = 11–12 yrs, and 7 = 13 yrs or older. Another group of 185 undergraduate or graduate students participated in other rating tasks for these pictures on 5-point scales: 36 rated concept familiarity, 36 image agreement, 37 visual complexity, 41 image variability, and 35 subjective word frequency. The data from six raters (i.e., five for image variability, and one for subjective word frequency) were excluded from further analysis due to their low consistency with those from other participants (i.e., the correlation coefficients between each participant's value and average of the whole group's on item were not significant). We followed the procedures and instructions by Alario and Ferrand [31] except for subjective word frequency for which we used the instructions from Morrison et al. [9]. In the concept familiarity task, the participants were asked to judge the familiarity of the concept of each picture according to their own experience. They were told to rate the concept itself, rather than the way it was drawn, on a 5-point scale (1 = a very unfamiliar object, 5 = a very familiar object). In the image agreement task, they were asked to judge how closely each picture resembled their mental image of the object (1 = very low agreement, 5 = very high agreement). In the visual complexity task, they were asked to rate the complexity of each drawing (1 = very simple, 5 = very complex). The image variability task required the participants to rate whether the dominant name of the object evoked few or many different images for that particular object (1 = few images, 5 = many images). The subjective word frequency task required the raters to estimate the frequency they encountered the dominant name of the object, either in speech or in writing form, on a scale from 1 to 5, where 1 = less than once a year, 2 = more than once a year but less than once a month, 3 = more than once a month but less than once a week, 4 = more than once a week but less than once a day, and 5 = at least once a day.

Unlike the Morrison et al. [9] study in which a child was tested on all the pictures, a child in the present study was only tested on part of the 435 pictures and the test session for each child was less than two hours. We assigned the 435 pictures to three subgroups by the difficulty level according to the rating results from three kindergarten teachers and undergraduates to reduce testing time. Seven age groups of children ranging from preschool to elementary school were tested (please see Table 1 for more details). Three critical groups were selected as starting points - kindergarten level 1, kindergarten level 3 and elementary school grade 2. If the accuracy of a picture was lower than 75% for a given group, the picture was tested on the next older group. If the accuracy was higher than 75%, the picture was tested on a younger age group. This procedure resulted in different number of children across groups, given that the number of pictures assigned to each group was different. Eventually, the AoA value for each picture was calculated from 18 to 25 children. Each picture was printed on a 5×8 cm paper as a card. The order of the pictures was randomized during testing. The children were tested individually in a quiet room in their schools. They were told that they were going to see pictures of objects, and their task was to name each picture, but some of the pictures were difficult and they might not recognize them or know the name. The testing procedure was very similar to Morrison et al. [9]. Participants were shown a picture and the experimenter asked them “What is this drawing?” (“zhe4 shang4mian4 hua4de shi4 shen2me?” in Chinese) They had approximately 5 sec to make a response, and if their reply was anything other than the target response, the experimenter told them to try again and cued them with the initial phoneme of the target name or the name of a related object. If they did not respond within 5 sec the experimenter moved on to the next picture.

In the picture naming experiment, the participants were tested one at a time in an experimental room. A fixation “+” was presented on the computer screen for 500 ms, followed by a picture in white against black background until the participant responded. There was a 2.5 sec timeout period before the next trial began. The responses and latency were recorded by the DMDX software [32]. The participants were instructed to name the picture as quickly and as accurately as possible into an external microphone that was connected to the computer. Each participant received all the 435 pictures in a randomized order. Practice stimuli were given before the experimental trial began. Three short breaks were included to prevent subject fatigue. The whole experimental session lasted about 75 minutes.

### Data analyses

#### Objective AoA - Children data

The scoring and acquisition criteria (75% rule) were similar to those used by Morrison et al. [9]. However, the calculation of the age when a picture was acquired by the 75% rule was modified using the formula Age  =  age_m – 4*(C% - 0.75), where *age_m* refers to the mean age in year of the youngest group of children who correctly named the picture above 75%, *C%* refers to the actual percentage of the children who named the picture correctly in this group. According to this formula, an item that is named correctly by 75% children with mean age of 8 years will receive an AoA score of 8 years; but an item named correctly by 100% children with mean age of 8 years will receive an AoA score of 7 years. Although this adjusted formula is not perfect, it represents our effort to make objective AoA a continuous variable and also to differentiate the items named correctly between 75% and 100% even if the two subgroups belong to the same chronological age group. Similarly, the AoA values for those pictures below 75% named correctly by the eldest group, the third grade students, were adjusted by the formula Age  =  9.27+4*(0.75- C%), where the number 9.27 is the mean age of the third grade students.

#### Name agreement

Following Snodgrass and Vanderwart [20], two measures were used for name agreement – the information statistic *H* and the percentage of subjects giving the most common name. The aforementioned three categories of naming failures - DKOs, DKNs, and TOTs - were eliminated when computing *H* values, but were included when computing the percentage agreement scores. *H* value was calculated for each picture by the formula
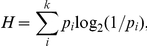



Where *k* refers to the number of different names given to each picture, *p*_i_ is the proportion of subjects giving each name.

Routinely, rated age of acquisition (AoA), subjective word frequency, concept agreement, concept familiarity, image agreement, image variability, and visual complexity, were calculated from the rated values averaged over participants. Concept agreement was operationally defined as the proportion of participants who correctly produced a name that reflects the concept of the picture. Word length referred to the number of character for the target name of the picture.

Naming responses from each participant and each picture were checked by two experimenters off line. The data from four participants were excluded from further analyses due to their high naming error rates (above 15%). Data with no responses, those named with a wrong concept, or those triggered by a cough were considered as invalid responses (5.6%) and were excluded from further analysis. Those data that fell beyond 2.5 SD of grand mean of naming latency (2.3%) were also excluded. Harmonic means of naming latency were calculated over participants for each picture with the remaining valid data. Averaged data for all variables were calculated across participants for each picture (see Table S1). Z scores were calculated for each variable for further analysis. We conducted Pearson correlation analysis and principal component factor analysis to explore the relationship between the various variables. Finally, we ran simultaneous multiple regression analyses with the variables as independent variables on naming latency to identify the influential variables during Chinese picture naming, including hierarchical multiple regression analyses to directly compare the relative contributions of objective AoA and rated AoA. In the final section, we will compare our data with existing data from other languages to identify cross-cultural and cross-linguistic differences.

## Results

The descriptive statistical data for all the variables and naming latencies are shown in Table 2. The grand mean of naming latency for all items was 1044 msec, which is very compatible with the results from Weekes et al. [7] (1025 msec) for Chinese speakers. The standardized residual plots show that the distribution of RT is acceptably normal (see also the skewness value in Table 2). Item-based indices were given in Table S1.

**Table 2:** Summary statistics for the picture naming latency and the 11 variables (N = 435).

Variable	Code	M	SD	Min.	Max.	Skewness
Naming latency	RT	1044	210	646	1809	0.64
Word length	Len	2.03	0.54	1	4	0.19
Image variability	Img_V	2.97	0.36	1.95	4.12	0.21
Image agreement	Img_A	3.87	0.47	2.22	4.81	−0.63
Concept familiarity	Fam	4.35	0.47	2.39	5.00	−1.20
Visual complexity	Vis_C	2.81	0.84	1.03	4.89	0.16
Subjective frequency	Freq_r	2.78	0.79	1.39	4.63	0.47
Name agreement (%)	NA%	0.66	0.23	0.08	1.00	−0.28
Name agreement (*H*)	H	1.32	0.84	0	4.29	0.37
Concept agreement	Cpt_A	0.86	0.16	0.18	1.00	−1.34
Rated AoA	AoA_r	3.44	1.14	1.24	6.87	0.40
Objective AoA	AoA_o	6.46	3.01	1.94	11.00	0.24

Note: RT was measured in millisecond, and word length in number of character.

Correlation coefficients between predicting variables and naming latency are shown in Table 3. Compared to Weekes et al. [7], the absolute values of coefficients between all the variables and RTs in the present study are larger. This discrepancy might be due to the larger number of items in the present study, which stabilises the relationship between variables. The data showed that the variables with the highest correlations with naming RTs are concept familiarity, followed by concept agreement, objective AoA, name agreement and rated AoA. In our data, objective AoA is no less correlated with other variables than rated AoA as suggested by some studies [9], [14], [15]. Both AoA indices correlate with concept familiarity and subjective frequency to a comparable level. However, the correlations with image agreement, name agreement and concept agreement are somewhat higher for objective AoA than for rated AoA. The correlation between the two AoA indices reached .502, which was similar to .558 in Alvarez and Cuetos [14] but not as high as .8 in Morrison et al. [9]. Obviously, the correlation between the two variables and name agreement is very high (.911), suggesting that the two variables almost measured the same thing. Other correlations between the variables are moderately high (*r*s<.800).

**Table 3:** Correlations between naming latency and 11 variables (n = 435).

	RT	Len	Img_V	Img_A	Fam	Vis_C	Freq_r	NA%	H	Cpt_A	AoA_o
Len	.132**										
Img_V	−.206***	−.097*									
Img_A	−.420***	.032	−.016								
Fam	−.757***	−.062	.192***	.442***							
Vis_C	.147***	.117*	−.045	−.087	−.197***						
Freq_r	−.430***	−.159***	.312***	.028	.471***	−.261***					
NA%	−.488***	−.076	.038	.392***	.412***	.013	.137**				
H	.424***	.042	.001	−.418***	−.331***	−.029	−.092	−.911***			
Cpt_A	−.664***	−.172***	.166***	.379***	.657***	−.070	.334***	.605***	−.522***		
AoA_o	.591***	.177***	−.183***	−.269***	−.476***	.134**	−.454***	−.421***	.387***	−.581***	
AoA_r	.475***	.315***	−.243***	−.048	−.392***	.263***	−.472***	−.232***	.182***	−.340***	.502***

Note:

* p<0.05,

** p<0.01;

*** p<0.001 (two-tailed).

The codes of variables are the same as in Table 2.

The factor analysis provides us with more information about the internal structure among these variables. The results are shown in Table 4. Six factors were extracted with the method of varimax rotation (i.e., maximizing the sum of the variance of the squared loadings) from a total of eleven variables: (1) *lexicon*, with high loadings on subjective frequency, objective AoA, rated AoA and concept agreement; (2) *name agreement*, with high loadings on H value, the percentage of name agreement, and concept agreement; (3) *semantics*, with high loadings on image agreement and concept familiarity; (4) *word length*, with high loadings only on the variable word length; (5) *visual complexity*, with high loadings only on the variable visual complexity; and (6) *image variability*, with high loadings only on the variable image variability. The six factors together accounted for 84.33% of the total variance, of which *lexicon* accounted for 22.21%, *name agreement* 20.02%, *semantics* 13.84%, *word length* 9.73%, *visual complexity* 9.32%, and *image variability* 9.21%. We can also observe from Table 5 that concept agreement loaded highly on the *lexicon*, *name agreement*, and *semantics* factors, and concept familiarity loaded highly on the *lexicon* and *semantics* factors.

**Table 4:** Rotated loadings on six factors for eleven variables.

Variable	Factor					
	Lexicon	Nameagreement	Semantics	Wordlength	Visualcomplexity	Imagevariability
Freq_r	.807	−.050	.012	.034	−.155	.203
AoA_o	−.728	−.316	−.191	.092	−.036	−.003
AoA_r	−.707	−.147	.089	.308	.219	−.085
Cpt_A	.521	.458	.480	−.117	.118	.059
H	−.098	−.955	−.165	.001	.000	.014
NA%	.181	.935	.198	−.023	.026	.007
Img_A	−.045	.244	.878	.025	−.092	−.014
Fam	.602	.142	.635	.031	−.053	.059
Len	−.135	−.015	.009	.972	.037	−.041
Vis_C	−.174	.037	−.084	.045	.961	.001
Img_V	.184	−.001	.017	−.047	.001	.978

Note: The variable codes are the same as in Table 2.

**Table 5:** Simultaneous multiple regression analyses on naming latency.

Variable	Standardized Beta	t Value	Tolerance	VIF
Fam	−0.461	−10.893***	0.425	2.352
AoA_o	0.160	4.164***	0.518	1.931
AoA_r	0.138	3.832***	0.586	1.708
Cpt_A	−0.131	−2.903**	0.376	2.656
NA%	−0.084	−2.326*	0.586	1.706
Img_A	−0.084	−2.528*	0.696	1.436
Img_V	−0.029	−0.974	0.885	1.129
Vis_C	−0.025	−0.862	0.872	1.146
Freq_r	−0.015	−0.405	0.589	1.698
Len	0.002	0.950	0.868	1.151

Note:

*p<0.05,

**p<0.01;

***p<0.001.

The variable codes are the same as in Table 2.

Because of the extremely high correlation between the percentage agreement scores and the H value, only one of them could be used in the multiple regression analysis at one time to avoid the multicollinearity problem. Simultaneous multiple regression analyses on naming RTs were run to explore the significant predictors of picture naming. The results were shown in Table 5. The VIF (i.e., variance inflation factor) and tolerance values suggest that the regression is not much affected by multicollinearity [33]. The total adjusted *R^2^* was 0.67, *F* (10, 424)  =  88.953, *p*<.001, which was twice that reported by Weekes et al. [7]. The variables showing significant contributions to naming RT were, in the order of decreasing standardized beta coefficients, concept familiarity, objective AoA, rated AoA, concept agreement, the percentage of name agreement, and image agreement. None of the other four variables, including subjective word frequency, was significant. When using H value as the index of name agreement in multiple regression analysis, we found almost the same results as discussed, indicating that either of these two indices - the percentage or H value was suitable to predict the latency of picture naming.

When comparing the relative contributions of objective AoA and rated AoA during picture naming task, eight other variables, including concept familiarity, subjective word frequency, image agreement, image variability, the percentage of name agreement, concept agreement, word length, and visual complexity, were first entered as predictors in the regression analysis. They explained 63.6% of the total variance of naming latency. Then objective AoA or rated AoA was added in the second step. Finally, objective AoA explained an additional 2.4% variance [*F* (1, 425) = 29.889, *p*<.001] and rated AoA 2.1% [*F* (1, 425) = 27.157, *p*<.001]. This result indicates that objective AoA and rated AoA have comparable predicting power for picture naming RTs, which disconfirms the idea that the objective AoA was a more powerful determinant of naming latency than rated AoA (see more in Discussion).

We compared our data with those from Dutch [34], English and Mandarin Chinese used in Taiwan (see [29]). Naming latency, name agreement in percentage and in H statistics, and concept agreement were given in Table 6.

**Table 6:** Comparison across languages on naming latency, name agreement, and concept agreement (N = 218).

	Mandarin_Beijing	Dutch	English	Mandarin_Taiwan
Naming latency				
Mean	1070	998	950	1106
SD	234	186	215	272
H-statistics				
Mean	1.22	.70	.47	.90
SD	.80	.60	.54	.71
NA %				
Mean	.69	.84	.90	.79
SD	.22	.19	.14	.21
Concept agreement				
Mean	.89	.92	.94	.88
SD	.16	.16	.11	.17

Note: The data for concept agreement for Dutch, English, and Taiwan Mandarin were calculated based on the sum of the percentages of the dominant name, morphological variants, and synonyms, which is identical to what Severens et al. [34] called “the lenient name agreements”.

ANOVA on naming latency showed significant differences among the four languages [*F* (3, 654) = 59.712, *p*<.001]. Pairwise comparison with Sidak adjustment [35] indicated that the average naming latency decreased gradually from Taiwan Mandarin to Beijing Mandarin, Dutch and English (all *p*s<.05). ANOVA on both H-statistics and name agreement in percentage also indicated significant differences among the four languages [*F* (3, 654) = 83.839, *p*<.001 for H-statistics, and *F* (3, 654) = 74.319, *p*<.001 for name agreement in percentage]. The H -statistics increased gradually from English to Dutch, Taiwan Mandarin, and Beijing Mandarin (all *p*s<.001). Reversely, the name agreement in percentage decreased gradually from English, Dutch, Taiwan Mandarin, and Beijing Mandarin (all *p*s<.01). Consistently with previous studies, name agreement (both in H-statistics and in percentage) in Chinese was lower than that in English or in Dutch [6], [34]. Interestingly, Beijing Mandarin showed much lower name agreement than Taiwan Mandarin. However, concept agreement in the two types of Mandarin was comparable, and comparable with that in Dutch (all *p*s >.1), which was lower than that in English (*p*<.001).

## Discussion

The present study is the first large-scale norming study of picture naming in Chinese. Twelve variables, including objective and rated AoA, the percentage of name agreement, H value, concept agreement, concept familiarity, subjective word frequency, image agreement, image variability, visual complexity, word length and naming latency, were made available for 435 line-drawing object pictures. Additionally, the regression analyses showed that in the current picture set, concept familiarity, objective AoA, rated AoA, concept agreement, name agreement, and image agreement were significant predictors of picture naming latency, whereas subjective frequency, word length, visual complexity, and image variability were not. Together, all these variables explained a large portion of the total variance (67%).

As mentioned in the Introduction, previous studies consistently found that rated AoA and name agreement had significant effects on picture naming. Results from our study confirm this observation. Moreover, objective AoA, which was less explored in previous studies, showed a comparable predictive power for picture naming latency as rated AoA. These patterns suggest that AoA (objective or rated) and name agreement (the percentage or H value) are key factors in picture naming across languages. In contrast to Chalard et al. 's [13] data from French that suggested that objective AoA was a more powerful determinant of naming latencies than rated AoA, we found that objective AoA and rated AoA accounted for similar amounts of variance after eight other variables were considered. In addition, we found that concept familiarity was the strongest predictor of all variables. This result is consistent with Weekes et al. [7] who reported a significant effect of concept familiarity, but differs from other studies suggesting that rated AoA was contaminated by familiarity [9], [14], [15]. The Table 1 in Juhsaz [4] suggested that concept familiarity seldom had a significant impact on picture naming when AoA was controlled.

Instead of using printed word frequency of picture names as done in Weekes et al. [7], the present study used subjectively rated word frequency and a larger number of pictures. However, neither study observed a significant frequency effect on picture naming. Lack of an effect of printed word frequency has also been found in many other studies when AoA was controlled for (see [4] for a review). It should be noted that, although there is no effect of subjective frequency, a large effect of concept familiarity exists in our data. Many previous studies have suggested that word frequency and concept familiarity effects have different origins in the cognitive model of word production [36] and involve different brain mechanisms [3]. Concept familiarity effects have been considered to originate in the phase of object identification whereas word frequency effects are assumed to mainly localize at phonological lexicon access [36], [37]. However, there have been studies that also suggest that word frequency and concept familiarity are difficult to disentangle and could take effect at the same phase (i.e., object identification) during the process of picture naming [6], [38]. If this latter suggestion is true, it could account for why many studies, including ours, observe concept familiarity effect but not word frequency effect.

Finally, unlike previous studies of English, Dutch, and Mandarin Chinese used in Taiwan [6], [34], the current study showed a lower name agreement and a higher H-statistic. Weekes et al. [7] reported name agreement (0.65) in Beijing Chinese consistent with the present study. On the other hand, comparable concept agreement has been found among Beijing, Taiwan Mandarin, and Dutch, suggesting that the concepts of these pictures are almost on the same familiarity level for the three groups of language users. Considering both the lower name agreement and a comparable concept agreement, we suggest that it is likely that there are more possible names referring to a given object picture for Mainland Chinese speakers compared to speakers of other languages or regions. The availability of more names for the same object might also be a cause for the generally slower naming latency in Mandarin Chinese than in other languages. While we do not have a definitive answer to why there is the discrepancy between Mandarin Chinese and other languages with regard to name agreement, our speculation is that Mandarin Chinese, especially that used in Mainland China, is often a mixture from speakers of many different dialects, as well as many ethnic groups. Although our participants were recruited as native Chinese speakers, they often had previous dialectal backgrounds and had been in close contact with speakers of different dialects at home or in school. Factors such as these are difficult to control in a large-scale experiment like ours but should be taken into consideration when explaining the research findings.

## Supporting Information

Table S1 Item-based indices for all 435 pictures.
(DOC)

Files S1 A subset of 169 pictures in zipped format.
(RAR)
