# Supplementary material for: Timed Picture Naming Norms for Mandarin Chinese
Source: PLoS One. 2011 Jan 26;6(1):e16505. doi: 10.1371/journal.pone.0016505 (PMC3027682; doi:10.1371/journal.pone.0016505)
Supplement: Table S1 — Item-based indices for all 435 pictures. (DOC) [file pone.0016505.s001.doc]

Table S1 item-based indices for all 435 pictures

| Name | English | Source | LEN | VAR | IMG | FAM | VC | FREQ | NA | H value | CA | AoA_r | AoA_o | error | RT_harm |
| --- | --- | --- | --- | --- | --- | --- | --- | --- | --- | --- | --- | --- | --- | --- | --- |
| 算盘 | abacus | 4 | 2 | 3.36 | 4.33 | 4.64 | 3.68 | 1.71 | 0.88 | 0.54 | 1.00 | 3.44 | 5.74 | 0.03 | 894 |
| 水母 | acaleph | 4 | 2 | 2.62 | 3.37 | 2.67 | 3.84 | 1.60 | 0.25 | 2.72 | 0.28 | 6.25 | 10.86 | 0.34 | 1635 |
| 手风琴 | accordion | 1 | 3 | 3.21 | 4.00 | 4.14 | 3.89 | 1.94 | 0.78 | 0.64 | 0.93 | 4.00 | 10.50 | 0.06 | 1102 |
| 飞机 | air plane | 1 | 2 | 3.31 | 4.28 | 4.67 | 2.62 | 2.97 | 0.90 | 0.55 | 1.00 | 2.76 | 2.25 | 0.00 | 748 |
| 闹钟 | alarm clock | 5 | 2 | 2.86 | 4.11 | 4.75 | 3.86 | 3.97 | 0.85 | 0.71 | 1.00 | 3.81 | 3.41 | 0.03 | 818 |
| 鳄鱼 | alligator | 1 | 2 | 2.69 | 4.31 | 4.17 | 3.86 | 2.09 | 0.90 | 0.62 | 0.95 | 4.18 | 4.42 | 0.00 | 1050 |
| 救护车 | ambulance | 4 | 3 | 2.60 | 3.72 | 4.50 | 2.97 | 2.43 | 0.93 | 0.45 | 0.98 | 4.31 | 4.66 | 0.03 | 1091 |
| 锚 | anchor | 1 | 1 | 1.95 | 2.96 | 3.50 | 2.11 | 1.94 | 0.45 | 1.95 | 0.48 | 5.63 | 11.00 | 0.37 | 1423 |
| 蚂蚁 | ant | 1 | 2 | 3.10 | 3.42 | 3.92 | 3.43 | 2.34 | 0.58 | 1.71 | 0.58 | 2.40 | 4.58 | 0.11 | 1282 |
| 天线 | antenna | 5 | 2 | 2.88 | 3.40 | 4.36 | 2.24 | 2.46 | 0.43 | 1.80 | 0.55 | 3.88 | 10.80 | 0.09 | 1148 |
| 苹果 | apple | 1 | 2 | 3.05 | 4.03 | 4.81 | 1.62 | 3.86 | 0.95 | 0.17 | 0.95 | 1.94 | 2.25 | 0.00 | 1016 |
| 手臂 | arm | 1 | 2 | 2.62 | 4.11 | 4.92 | 2.05 | 3.69 | 0.53 | 2.15 | 1.00 | 2.50 | 3.56 | 0.00 | 895 |
| 箭 | arrow | 5 | 1 | 2.81 | 3.45 | 4.44 | 1.38 | 2.14 | 0.63 | 1.31 | 0.78 | 3.31 | 4.72 | 0.00 | 1062 |
| 箭头 | arrow | 1 | 2 | 2.83 | 4.17 | 4.61 | 1.19 | 2.88 | 0.73 | 1.60 | 1.00 | 4.38 | 5.19 | 0.00 | 848 |
| 烟灰缸 | ashtray | 1 | 3 | 3.12 | 3.75 | 3.61 | 2.08 | 2.34 | 0.43 | 2.18 | 0.48 | 3.94 | 10.70 | 0.26 | 1390 |
| 斧头 | axe | 1 | 2 | 3.29 | 4.49 | 4.78 | 1.76 | 1.94 | 0.73 | 1.04 | 1.00 | 3.12 | 5.77 | 0.03 | 860 |
| 羽毛球 | badminton | 4 | 3 | 3.38 | 4.63 | 4.92 | 3.68 | 3.03 | 1.00 | 0.00 | 1.00 | 4.18 | 4.58 | 0.00 | 839 |
| 书包 | bag | 5 | 2 | 2.95 | 3.06 | 4.75 | 2.43 | 4.31 | 0.60 | 2.03 | 1.00 | 2.56 | 4.66 | 0.00 | 866 |
| 气球 | ball | 1 | 2 | 3.07 | 4.42 | 4.69 | 1.14 | 2.80 | 0.95 | 0.17 | 0.95 | 2.00 | 2.81 | 0.06 | 1007 |
| 竹子 | bamboo | 5 | 2 | 2.55 | 3.97 | 4.72 | 2.05 | 2.29 | 0.73 | 0.91 | 0.93 | 3.31 | 4.58 | 0.00 | 975 |
| 笋 | bamboo sprouts | 5 | 1 | 2.57 | 3.74 | 3.69 | 4.27 | 2.31 | 0.35 | 1.72 | 0.70 | 4.47 | 10.35 | 0.03 | 1231 |
| 香蕉 | banana | 1 | 2 | 3.12 | 4.08 | 4.72 | 1.43 | 3.68 | 1.00 | 0.00 | 1.00 | 2.18 | 2.63 | 0.06 | 906 |
| 乐队 | band | 2 | 2 | 2.73 | 3.63 | 4.36 | 4.73 | 2.23 | 0.88 | 0.63 | 0.93 | 5.38 | 10.33 | 0.00 | 1182 |
| 篮子 | basket | 1 | 2 | 3.45 | 3.64 | 4.42 | 3.83 | 2.66 | 0.63 | 2.00 | 0.98 | 2.25 | 4.73 | 0.00 | 924 |
| 篮球 | basket-ball | 5 | 2 | 2.95 | 3.88 | 4.53 | 1.54 | 3.66 | 0.75 | 0.95 | 0.75 | 4.25 | 8.27 | 0.03 | 1068 |
| 蝙蝠 | bat | 1 | 2 | 2.36 | 4.17 | 4.11 | 3.11 | 2.00 | 0.98 | 0.00 | 0.98 | 4.00 | 5.58 | 0.03 | 1022 |
| 浴缸 | bathtub | 4 | 2 | 2.79 | 4.17 | 4.39 | 2.24 | 2.37 | 0.50 | 2.18 | 0.90 | 5.31 | 5.68 | 0.03 | 1076 |
| 熊 | bear | 1 | 1 | 2.93 | 3.61 | 3.89 | 3.19 | 2.65 | 0.45 | 1.40 | 0.90 | 4.47 | 10.45 | 0.03 | 1221 |
| 床 | bed | 1 | 1 | 3.00 | 4.00 | 4.89 | 2.65 | 4.51 | 0.88 | 0.63 | 1.00 | 1.94 | 2.84 | 0.00 | 710 |
| 蜜蜂 | bee | 1 | 2 | 3.43 | 3.33 | 3.75 | 3.78 | 2.60 | 0.38 | 2.55 | 0.45 | 2.35 | 10.32 | 0.03 | 1282 |
| 甲虫 | beetle | 1 | 2 | 3.14 | 3.22 | 3.56 | 2.81 | 2.06 | 0.13 | 3.30 | 0.45 | 3.81 | 10.59 | 0.20 | 1459 |
| 铃铛 | bell | 1 | 2 | 2.24 | 3.97 | 4.61 | 2.46 | 2.26 | 0.40 | 1.93 | 0.68 | 2.94 | 4.41 | 0.03 | 1022 |
| 皮带 | belt | 1 | 2 | 3.02 | 4.22 | 4.75 | 1.97 | 3.49 | 0.53 | 1.77 | 0.85 | 3.35 | 4.69 | 0.00 | 993 |
| 自行车 | bike | 1 | 3 | 3.43 | 4.33 | 4.94 | 3.57 | 4.17 | 0.98 | 0.17 | 1.00 | 2.81 | 2.81 | 0.00 | 763 |
| 打气筒 | bicycle pump | 1 | 3 | 2.88 | 4.26 | 4.25 | 1.86 | 3.14 | 0.58 | 1.78 | 0.88 | 3.50 | 4.68 | 0.06 | 1202 |
| 望远镜 | binocular | 1 | 3 | 3.07 | 4.17 | 3.97 | 3.78 | 2.26 | 0.73 | 0.18 | 0.95 | 4.18 | 7.17 | 0.03 | 1063 |
| 小鸟 | bird | 1 | 2 | 3.55 | 4.22 | 4.47 | 2.65 | 3.37 | 0.53 | 2.01 | 0.95 | 1.56 | 2.74 | 0.09 | 972 |
| 鸟巢 | bird's nest | 1 | 2 | 2.79 | 3.97 | 3.78 | 4.62 | 2.31 | 0.43 | 1.69 | 0.83 | 3.00 | 7.00 | 0.06 | 1537 |
| 血 | blood | 2 | 1 | 2.81 | 3.20 | 3.61 | 3.43 | 3.17 | 0.50 | 2.40 | 0.73 | 1.81 | 6.25 | 0.06 | 1411 |
| 上衣 | blouse | 1 | 2 | 3.29 | 4.08 | 4.81 | 2.46 | 3.94 | 0.23 | 3.37 | 1.00 | 3.44 | 10.91 | 0.03 | 959 |
| 船 | boat | 4 | 1 | 3.71 | 3.56 | 4.50 | 3.73 | 2.57 | 0.53 | 2.30 | 0.98 | 2.56 | 4.20 | 0.00 | 1026 |
| 骨头 | bone | 2 | 2 | 3.26 | 4.61 | 4.33 | 2.16 | 2.77 | 0.95 | 0.34 | 1.00 | 2.50 | 3.18 | 0.03 | 910 |
| 书 | book | 1 | 1 | 3.62 | 4.40 | 4.83 | 2.22 | 4.63 | 0.88 | 0.71 | 1.00 | 1.88 | 2.84 | 0.00 | 799 |
| 书架 | bookshelf | 4 | 2 | 3.02 | 3.81 | 4.47 | 2.67 | 3.69 | 0.65 | 1.44 | 0.95 | 3.71 | 7.09 | 0.00 | 1088 |
| 靴子 | boot | 1 | 2 | 2.81 | 3.63 | 4.75 | 2.24 | 2.76 | 0.65 | 2.05 | 0.88 | 4.59 | 7.45 | 0.00 | 920 |
| 瓶子 | bottle | 1 | 2 | 3.69 | 4.14 | 4.75 | 1.51 | 3.23 | 0.45 | 1.81 | 1.00 | 2.35 | 3.67 | 0.00 | 853 |
| 弓 | bow | 4 | 1 | 2.71 | 3.74 | 4.14 | 2.11 | 1.94 | 0.83 | 0.63 | 0.90 | 3.56 | 8.27 | 0.00 | 1069 |
| 蝴蝶结 | bowknot | 1 | 3 | 3.36 | 3.83 | 4.61 | 2.14 | 2.29 | 0.70 | 1.10 | 0.93 | 3.25 | 5.78 | 0.03 | 907 |
| 碗 | bow | 1 | 1 | 2.74 | 4.14 | 4.69 | 1.59 | 3.97 | 1.00 | 0.00 | 1.00 | 1.56 | 2.48 | 0.00 | 782 |
| 盒子 | box | 1 | 2 | 2.83 | 3.60 | 4.06 | 1.22 | 3.69 | 0.50 | 2.47 | 0.65 | 2.06 | 5.68 | 0.06 | 1083 |
| 大脑 | brain | 5 | 2 | 3.05 | 4.26 | 3.81 | 4.11 | 3.62 | 0.65 | 1.35 | 0.95 | 4.06 | 10.36 | 0.00 | 1017 |
| 面包 | bread | 1 | 2 | 3.93 | 3.75 | 4.42 | 1.84 | 4.03 | 0.75 | 1.07 | 0.90 | 3.25 | 3.56 | 0.03 | 973 |
| 桥 | bridge | 4 | 1 | 3.05 | 3.08 | 4.42 | 3.08 | 3.11 | 0.53 | 2.19 | 1.00 | 2.71 | 3.73 | 0.06 | 1053 |
| 刀 | broadsword | 5 | 1 | 2.74 | 3.33 | 4.50 | 2.14 | 3.43 | 0.58 | 1.47 | 0.95 | 2.50 | 7.09 | 0.03 | 881 |
| 扫帚 | broom | 1 | 2 | 3.21 | 3.92 | 4.72 | 2.73 | 3.54 | 0.43 | 2.19 | 0.80 | 2.20 | 6.94 | 0.06 | 1038 |
| 刷子 | brush | 1 | 2 | 2.93 | 3.79 | 4.58 | 2.65 | 2.89 | 0.83 | 0.97 | 0.95 | 3.00 | 10.45 | 0.03 | 898 |
| 水桶 | barrel | 1 | 2 | 3.36 | 3.75 | 4.31 | 2.65 | 2.91 | 0.55 | 1.77 | 0.98 | 2.41 | 5.67 | 0.00 | 1022 |
| 子弹 | bullet | 5 | 2 | 2.48 | 3.47 | 4.25 | 3.03 | 2.20 | 0.80 | 0.83 | 0.85 | 3.63 | 5.74 | 0.09 | 1191 |
| 公交车 | bus | 1 | 3 | 3.36 | 4.31 | 4.94 | 3.54 | 4.17 | 0.43 | 2.40 | 1.00 | 5.25 | 3.19 | 0.00 | 1026 |
| 蝴蝶 | butterfly | 1 | 2 | 2.90 | 4.58 | 4.78 | 3.43 | 2.29 | 0.98 | 0.17 | 0.98 | 2.88 | 1.94 | 0.00 | 727 |
| 钮扣 | button | 1 | 2 | 3.33 | 4.44 | 4.25 | 1.65 | 3.51 | 0.50 | 1.13 | 0.85 | 2.65 | 3.56 | 0.09 | 1054 |
| 白菜 | cabbage | 5 | 2 | 2.95 | 3.79 | 4.22 | 4.27 | 3.65 | 0.75 | 1.34 | 0.90 | 2.13 | 3.67 | 0.06 | 920 |
| 仙人掌 | cactus | 4 | 3 | 2.88 | 3.11 | 3.97 | 3.27 | 1.91 | 0.73 | 0.91 | 0.75 | 3.69 | 5.73 | 0.11 | 1264 |
| 蛋糕 | cake | 1 | 2 | 3.10 | 3.50 | 4.56 | 2.75 | 3.29 | 0.88 | 0.18 | 0.88 | 3.06 | 3.56 | 0.03 | 973 |
| 日历 | calendar | 5 | 2 | 3.62 | 3.83 | 4.86 | 2.68 | 3.51 | 0.90 | 0.62 | 1.00 | 2.81 | 7.44 | 0.00 | 924 |
| 骆驼 | camel | 1 | 2 | 2.93 | 3.34 | 4.36 | 3.03 | 1.89 | 0.80 | 0.79 | 0.98 | 3.50 | 4.72 | 0.03 | 939 |
| 照相机 | camera | 1 | 3 | 3.48 | 4.17 | 4.72 | 3.78 | 3.23 | 0.60 | 0.97 | 1.00 | 4.13 | 3.78 | 0.00 | 876 |
| 蜡烛 | candle | 1 | 2 | 2.81 | 4.64 | 4.83 | 2.16 | 2.29 | 0.93 | 0.45 | 1.00 | 2.38 | 3.78 | 0.00 | 757 |
| 糖 | candy | 4 | 1 | 2.88 | 4.31 | 4.64 | 2.49 | 3.73 | 0.60 | 1.09 | 0.95 | 1.71 | 2.63 | 0.03 | 929 |
| 大炮 | cannon | 1 | 2 | 3.64 | 3.11 | 3.69 | 3.84 | 1.94 | 0.63 | 1.44 | 0.80 | 3.06 | 10.35 | 0.17 | 1136 |
| 帽子 | cap | 1 | 2 | 3.62 | 2.97 | 4.58 | 1.92 | 3.31 | 0.80 | 1.03 | 1.00 | 2.00 | 4.17 | 0.00 | 832 |
| 轿车 | car | 1 | 2 | 3.62 | 4.19 | 4.89 | 3.68 | 3.44 | 0.35 | 2.15 | 1.00 | 4.00 | 2.74 | 0.00 | 906 |
| 扑克 | card | 4 | 2 | 2.95 | 4.06 | 4.94 | 2.32 | 2.63 | 0.58 | 1.60 | 1.00 | 3.47 | 4.18 | 0.00 | 816 |
| 萝卜 | carrot | 1 | 2 | 3.07 | 3.94 | 4.78 | 2.54 | 3.24 | 0.50 | 1.14 | 1.00 | 2.53 | 4.60 | 0.00 | 839 |
| 猫 | cat | 1 | 1 | 3.07 | 3.75 | 4.75 | 2.30 | 3.11 | 0.98 | 0.17 | 0.98 | 1.88 | 2.86 | 0.03 | 839 |
| 弹弓 | catapult | 4 | 2 | 2.83 | 4.56 | 4.67 | 2.16 | 1.66 | 0.75 | 1.10 | 0.85 | 2.69 | 10.55 | 0.06 | 1178 |
| 毛毛虫 | caterpillar | 1 | 3 | 2.38 | 4.08 | 4.00 | 3.27 | 2.06 | 0.55 | 1.57 | 0.65 | 4.88 | 5.78 | 0.09 | 927 |
| 芹菜 | celery | 1 | 2 | 3.07 | 3.46 | 4.00 | 3.86 | 3.26 | 0.55 | 1.89 | 0.55 | 3.29 | 10.55 | 0.14 | 1168 |
| 大提琴 | cello | 4 | 3 | 2.36 | 3.71 | 4.36 | 3.49 | 2.06 | 0.45 | 2.20 | 0.80 | 5.18 | 10.93 | 0.03 | 1003 |
| 铁链 | chain | 1 | 2 | 3.07 | 4.39 | 4.42 | 2.32 | 2.23 | 0.58 | 1.74 | 0.98 | 3.65 | 5.74 | 0.00 | 1047 |
| 椅子 | chair | 1 | 2 | 3.95 | 4.03 | 4.97 | 2.11 | 4.14 | 0.45 | 0.63 | 1.00 | 2.00 | 5.68 | 0.00 | 771 |
| 樱桃 | cherry | 1 | 2 | 2.69 | 4.00 | 4.19 | 1.70 | 2.51 | 0.55 | 1.56 | 0.55 | 4.12 | 10.68 | 0.06 | 1109 |
| 小鸡 | chicken | 2 | 2 | 2.69 | 4.03 | 4.33 | 3.08 | 2.23 | 0.83 | 0.90 | 0.83 | 2.06 | 10.45 | 0.03 | 1024 |
| 烟囱 | chimney | 1 | 2 | 2.88 | 4.06 | 4.67 | 3.19 | 2.23 | 0.98 | 0.17 | 0.98 | 3.18 | 4.69 | 0.00 | 858 |
| 教堂 | church | 1 | 2 | 3.24 | 3.25 | 4.08 | 3.19 | 2.09 | 0.40 | 2.09 | 0.43 | 5.94 | 11.00 | 0.03 | 1014 |
| 烟 | cigarette | 1 | 1 | 3.10 | 4.42 | 4.39 | 1.92 | 2.91 | 0.73 | 1.17 | 0.95 | 3.69 | 7.09 | 0.03 | 966 |
| 钟 | clock | 5 | 1 | 2.90 | 3.61 | 4.53 | 2.83 | 3.57 | 0.50 | 2.44 | 0.98 | 2.00 | 4.20 | 0.00 | 987 |
| 夹子 | cramp | 1 | 2 | 2.98 | 3.40 | 4.61 | 2.38 | 3.03 | 0.68 | 1.57 | 0.88 | 2.94 | 9.16 | 0.11 | 1122 |
| 云 | cloud | 5 | 1 | 2.88 | 3.28 | 4.36 | 1.86 | 3.43 | 0.45 | 2.16 | 0.98 | 2.19 | 3.73 | 0.00 | 988 |
| 小丑 | clown | 1 | 2 | 3.19 | 3.56 | 3.58 | 4.00 | 1.89 | 0.60 | 1.65 | 0.60 | 4.44 | 5.74 | 0.09 | 1289 |
| 大衣 | coat | 1 | 2 | 3.24 | 4.03 | 4.75 | 2.32 | 2.91 | 0.55 | 1.69 | 0.95 | 3.82 | 9.21 | 0.03 | 899 |
| 梳子 | comb | 1 | 2 | 2.95 | 3.42 | 4.75 | 2.35 | 4.26 | 0.98 | 0.17 | 1.00 | 2.59 | 4.20 | 0.00 | 778 |
| 指南针 | compass | 4 | 3 | 2.81 | 4.06 | 4.53 | 2.76 | 2.03 | 0.75 | 1.32 | 0.75 | 4.50 | 10.40 | 0.06 | 997 |
| 电脑 | computer | 5 | 2 | 3.12 | 4.36 | 4.97 | 4.78 | 4.54 | 1.00 | 0.00 | 1.00 | 5.81 | 2.81 | 0.03 | 873 |
| 玉米 | corn | 1 | 2 | 2.40 | 4.47 | 4.75 | 3.78 | 3.46 | 0.95 | 0.34 | 1.00 | 2.53 | 3.73 | 0.03 | 824 |
| 沙发 | couch | 1 | 2 | 2.71 | 4.08 | 4.86 | 2.14 | 3.23 | 0.93 | 0.50 | 0.98 | 4.25 | 3.42 | 0.03 | 878 |
| 牛 | cow | 1 | 1 | 3.12 | 3.94 | 4.67 | 3.68 | 2.66 | 0.68 | 1.57 | 0.98 | 3.76 | 7.71 | 0.00 | 899 |
| 螃蟹 | crab | 4 | 2 | 2.95 | 3.77 | 4.17 | 3.19 | 2.31 | 0.85 | 0.61 | 1.00 | 4.12 | 2.81 | 0.00 | 1051 |
| 仙鹤 | crane | 5 | 2 | 3.07 | 3.51 | 3.94 | 3.22 | 1.89 | 0.28 | 2.54 | 0.78 | 4.41 | 9.16 | 0.14 | 1300 |
| 皇冠 | crown | 1 | 2 | 2.90 | 3.67 | 3.67 | 3.51 | 1.83 | 0.48 | 1.54 | 0.80 | 4.06 | 5.77 | 0.03 | 1184 |
| 拐杖 | crutch | 3 | 2 | 2.57 | 3.03 | 4.42 | 2.68 | 2.11 | 0.73 | 0.88 | 0.90 | 3.53 | 10.45 | 0.03 | 1337 |
| 丝瓜 | cucumber | 5 | 2 | 3.19 | 4.39 | 4.14 | 3.89 | 2.31 | 0.53 | 1.28 | 0.53 | 3.50 | 10.85 | 0.11 | 1477 |
| 茶杯 | cup | 1 | 2 | 3.14 | 4.11 | 4.75 | 1.81 | 3.91 | 0.45 | 1.51 | 1.00 | 2.36 | 2.74 | 0.00 | 816 |
| 窗帘 | curtain | 4 | 2 | 3.19 | 3.31 | 4.44 | 2.54 | 3.63 | 0.85 | 0.64 | 0.93 | 2.88 | 3.58 | 0.09 | 1250 |
| 飞镖 | dart | 1 | 2 | 3.17 | 3.89 | 3.89 | 3.39 | 1.71 | 0.48 | 2.01 | 0.55 | 4.94 | 10.60 | 0.14 | 1502 |
| 鹿 | deer | 1 | 1 | 2.79 | 3.97 | 4.36 | 3.24 | 2.14 | 0.63 | 1.53 | 0.95 | 3.25 | 10.64 | 0.06 | 1021 |
| 书桌 | desk | 1 | 2 | 2.90 | 3.50 | 4.81 | 2.81 | 4.06 | 0.38 | 1.90 | 0.95 | 2.88 | 10.60 | 0.00 | 1087 |
| 钻石 | diamond | 2 | 2 | 2.93 | 4.06 | 3.86 | 2.42 | 2.14 | 0.68 | 0.81 | 0.70 | 4.65 | 10.33 | 0.06 | 1245 |
| 骰子 | dice | 3 | 2 | 2.90 | 4.35 | 4.50 | 3.08 | 1.94 | 0.75 | 0.89 | 0.75 | 4.76 | 9.16 | 0.00 | 1254 |
| 恐龙 | dinosaur | 3 | 2 | 2.79 | 3.50 | 3.83 | 3.11 | 2.17 | 0.88 | 0.36 | 0.93 | 4.00 | 3.73 | 0.09 | 1332 |
| 光盘 | disk | 2 | 2 | 3.24 | 4.61 | 4.81 | 2.38 | 3.38 | 0.53 | 2.07 | 0.98 | 6.06 | 4.84 | 0.03 | 1035 |
| 医生 | doctor | 5 | 2 | 3.02 | 2.69 | 4.61 | 4.08 | 3.20 | 0.80 | 0.98 | 1.00 | 2.63 | 4.42 | 0.00 | 1276 |
| 狗 | dog | 1 | 1 | 3.64 | 4.22 | 4.67 | 2.46 | 3.46 | 0.95 | 0.29 | 1.00 | 1.88 | 2.48 | 0.00 | 885 |
| 驴 | donkey | 1 | 1 | 2.71 | 3.71 | 4.39 | 3.46 | 2.11 | 0.50 | 2.02 | 0.70 | 2.94 | 8.77 | 0.00 | 1005 |
| 门 | door | 1 | 1 | 3.69 | 3.81 | 4.86 | 2.57 | 4.40 | 0.83 | 0.87 | 0.98 | 1.76 | 2.25 | 0.03 | 741 |
| 门把手 | door knob | 1 | 3 | 2.88 | 3.11 | 3.72 | 2.24 | 3.24 | 0.15 | 3.57 | 0.35 | 3.63 | 10.73 | 0.23 | 1558 |
| 龙 | dragon | 5 | 1 | 2.74 | 3.92 | 4.36 | 4.89 | 2.23 | 0.78 | 0.68 | 0.83 | 2.94 | 3.56 | 0.03 | 1009 |
| 蜻蜓 | dragon fly | 4 | 2 | 3.36 | 4.61 | 4.58 | 3.05 | 2.09 | 1.00 | 0.00 | 1.00 | 2.44 | 3.73 | 0.00 | 875 |
| 抽屉 | drawer | 4 | 2 | 2.79 | 3.97 | 4.69 | 1.95 | 3.86 | 0.85 | 0.70 | 0.95 | 2.94 | 7.89 | 0.06 | 994 |
| 连衣裙 | dress | 1 | 3 | 3.38 | 3.53 | 4.69 | 1.95 | 2.74 | 0.53 | 2.10 | 0.88 | 3.56 | 9.16 | 0.00 | 926 |
| 柜子 | dresser | 1 | 2 | 2.93 | 3.29 | 4.69 | 2.62 | 3.60 | 0.63 | 1.80 | 0.80 | 2.50 | 5.67 | 0.00 | 1167 |
| 电钻 | drill | 2 | 2 | 2.90 | 3.97 | 4.11 | 3.27 | 1.69 | 0.28 | 3.09 | 0.63 | 5.75 | 10.73 | 0.11 | 1248 |
| 鼓 | drum | 3 | 1 | 2.74 | 3.92 | 4.19 | 3.59 | 2.17 | 0.78 | 1.38 | 0.98 | 3.00 | 4.58 | 0.03 | 971 |
| 鸭子 | duck | 1 | 2 | 2.83 | 3.97 | 4.69 | 2.62 | 2.74 | 0.50 | 1.19 | 0.50 | 2.24 | 10.60 | 0.03 | 975 |
| 杠铃 | dumbbell | 2 | 2 | 3.12 | 3.84 | 4.39 | 2.92 | 2.06 | 0.50 | 1.62 | 0.58 | 5.35 | 10.60 | 0.23 | 1322 |
| 鹰 | eagle | 1 | 1 | 2.52 | 3.67 | 4.31 | 3.43 | 2.14 | 0.50 | 1.92 | 0.68 | 3.50 | 5.58 | 0.03 | 1158 |
| 耳朵 | ear | 1 | 2 | 2.33 | 4.47 | 4.97 | 2.57 | 3.80 | 0.90 | 0.57 | 1.00 | 1.94 | 3.73 | 0.00 | 696 |
| 耳环 | earring | 4 | 2 | 2.88 | 2.76 | 4.19 | 2.57 | 2.94 | 0.33 | 2.93 | 0.75 | 3.88 | 10.55 | 0.09 | 1265 |
| 蚯蚓 | earthworm | 1 | 2 | 3.14 | 3.69 | 3.75 | 2.32 | 1.89 | 0.68 | 1.27 | 0.70 | 2.88 | 9.03 | 0.14 | 1204 |
| 鸡蛋 | egg | 2 | 2 | 3.00 | 4.11 | 4.19 | 2.27 | 4.23 | 0.68 | 0.91 | 1.00 | 2.00 | 5.78 | 0.03 | 969 |
| 茄子 | eggplant | 5 | 2 | 2.74 | 3.53 | 4.25 | 2.27 | 3.26 | 0.70 | 1.09 | 0.73 | 2.63 | 5.67 | 0.00 | 1188 |
| 大象 | elephant | 1 | 2 | 2.64 | 4.42 | 4.58 | 3.76 | 2.17 | 0.88 | 0.63 | 1.00 | 2.81 | 2.63 | 0.00 | 785 |
| 信封 | envelope | 1 | 2 | 3.17 | 4.14 | 4.72 | 1.22 | 2.83 | 0.83 | 0.77 | 0.98 | 3.81 | 8.92 | 0.00 | 837 |
| 橡皮擦 | eraser | 2 | 2 | 2.60 | 3.34 | 4.36 | 1.57 | 1.82 | 0.45 | 1.90 | 0.60 | 2.60 | 7.17 | 0.03 | 1167 |
| 眼睛 | eye | 1 | 2 | 2.71 | 4.06 | 4.97 | 3.00 | 4.29 | 0.78 | 1.04 | 1.00 | 1.53 | 1.94 | 0.00 | 646 |
| 扇子 | fan | 5 | 2 | 2.86 | 3.97 | 4.64 | 3.95 | 2.76 | 0.78 | 1.23 | 1.00 | 2.33 | 3.67 | 0.00 | 842 |
| 羽毛 | feather | 5 | 2 | 2.95 | 4.09 | 4.17 | 2.27 | 2.66 | 0.83 | 1.06 | 0.95 | 2.65 | 6.94 | 0.00 | 996 |
| 栅栏 | fence | 1 | 2 | 3.14 | 4.36 | 4.36 | 2.05 | 2.23 | 0.78 | 1.05 | 0.98 | 4.12 | 8.67 | 0.03 | 1080 |
| 食指 | finger | 1 | 2 | 2.52 | 4.47 | 4.86 | 2.54 | 3.57 | 0.55 | 1.40 | 1.00 | 1.44 | 5.65 | 0.00 | 825 |
| 火 | fire | 5 | 1 | 3.07 | 3.50 | 3.67 | 3.54 | 3.43 | 0.48 | 1.67 | 0.88 | 1.75 | 5.19 | 0.03 | 1105 |
| 鱼 | fish | 1 | 1 | 3.14 | 4.14 | 4.72 | 3.46 | 3.57 | 0.73 | 1.40 | 1.00 | 2.18 | 2.63 | 0.00 | 704 |
| 鱼缸 | fish global | 1 | 2 | 2.88 | 3.88 | 4.36 | 3.70 | 2.40 | 0.70 | 1.53 | 0.88 | 3.75 | 3.78 | 0.06 | 1208 |
| 鱼钩 | fishhook | 1 | 2 | 3.02 | 4.09 | 3.97 | 1.59 | 2.09 | 0.23 | 1.96 | 0.58 | 3.69 | 10.75 | 0.11 | 1283 |
| 拳头 | fist | 2 | 2 | 2.62 | 4.47 | 4.75 | 3.03 | 3.29 | 0.88 | 0.71 | 1.00 | 2.56 | 6.69 | 0.00 | 835 |
| 红旗 | flage | 1 | 2 | 2.88 | 3.86 | 4.81 | 1.73 | 2.51 | 0.30 | 2.17 | 1.00 | 2.38 | 2.86 | 0.03 | 809 |
| 软盘 | floppy disk | 5 | 2 | 2.57 | 4.26 | 3.44 | 3.11 | 3.00 | 0.48 | 2.02 | 0.83 | 6.87 | 10.40 | 0.11 | 1573 |
| 花 | flower | 1 | 1 | 4.12 | 3.61 | 4.69 | 2.49 | 3.77 | 0.60 | 1.52 | 0.83 | 1.59 | 2.25 | 0.06 | 824 |
| 笛子 | flute | 1 | 2 | 2.79 | 3.66 | 2.94 | 2.95 | 2.14 | 0.25 | 2.52 | 0.63 | 3.81 | 5.77 | 0.20 | 1357 |
| 苍蝇 | fly | 1 | 2 | 2.81 | 3.69 | 4.03 | 3.32 | 2.83 | 0.63 | 1.86 | 0.70 | 2.44 | 10.45 | 0.06 | 1127 |
| 脚 | foot | 1 | 1 | 3.12 | 4.20 | 5.00 | 2.19 | 4.32 | 0.68 | 1.56 | 1.00 | 1.29 | 2.25 | 0.00 | 791 |
| 橄榄球 | football | 1 | 3 | 2.10 | 4.00 | 3.11 | 2.41 | 1.97 | 0.45 | 0.72 | 0.50 | 5.75 | 10.50 | 0.31 | 1374 |
| 叉子 | fork | 1 | 2 | 3.02 | 4.38 | 4.67 | 1.89 | 2.77 | 0.88 | 0.63 | 0.98 | 2.75 | 3.19 | 0.03 | 874 |
| 狐狸 | fox | 1 | 2 | 2.69 | 3.69 | 4.36 | 2.97 | 2.06 | 0.70 | 1.27 | 0.80 | 2.94 | 5.78 | 0.00 | 988 |
| 青蛙 | frog | 1 | 2 | 2.69 | 4.26 | 4.72 | 2.86 | 2.46 | 0.90 | 0.62 | 1.00 | 2.50 | 2.63 | 0.00 | 809 |
| 漏斗 | funnel | 1 | 2 | 2.76 | 4.16 | 4.50 | 1.22 | 2.14 | 0.98 | 0.17 | 0.98 | 4.06 | 10.40 | 0.03 | 1022 |
| 垃圾桶 | garbage | 1 | 3 | 3.56 | 3.58 | 4.47 | 2.86 | 4.14 | 0.48 | 1.95 | 0.53 | 4.00 | 4.20 | 0.03 | 1105 |
| 长颈鹿 | giraffe | 1 | 3 | 2.43 | 4.50 | 4.53 | 4.30 | 1.86 | 0.93 | 0.45 | 0.98 | 3.18 | 2.63 | 0.00 | 884 |
| 女孩 | girl | 4 | 2 | 3.71 | 3.47 | 4.78 | 2.73 | 4.29 | 0.60 | 2.12 | 0.93 | 2.19 | 7.84 | 0.00 | 913 |
| 杯子 | glass | 1 | 2 | 3.40 | 3.69 | 4.67 | 1.76 | 4.20 | 0.45 | 1.75 | 1.00 | 3.13 | 4.44 | 0.06 | 806 |
| 眼镜 | glasses | 1 | 2 | 3.71 | 4.44 | 4.97 | 2.08 | 4.17 | 1.00 | 0.00 | 1.00 | 3.71 | 2.25 | 0.00 | 694 |
| 地球 | globe | 4 | 2 | 2.52 | 4.00 | 4.81 | 3.35 | 3.09 | 0.58 | 1.12 | 1.00 | 3.38 | 4.42 | 0.00 | 844 |
| 手套 | glove | 1 | 2 | 3.17 | 3.97 | 4.75 | 2.62 | 3.12 | 0.93 | 0.50 | 0.95 | 2.81 | 2.69 | 0.00 | 751 |
| 山羊 | goat | 1 | 2 | 2.79 | 4.26 | 4.58 | 2.68 | 1.97 | 0.80 | 0.89 | 0.95 | 2.44 | 9.16 | 0.03 | 1049 |
| 金鱼 | goldfish | 5 | 2 | 3.67 | 3.32 | 3.53 | 4.35 | 2.34 | 0.85 | 0.64 | 0.90 | 3.25 | 7.94 | 0.03 | 1222 |
| 锣 | gong | 5 | 1 | 2.27 | 4.00 | 4.25 | 2.16 | 1.86 | 0.73 | 1.22 | 0.88 | 4.19 | 10.32 | 0.06 | 1142 |
| 鹅 | goose | 5 | 2 | 3.07 | 3.53 | 4.53 | 1.97 | 2.57 | 0.70 | 1.56 | 0.93 | 2.13 | 4.72 | 0.03 | 986 |
| 猩猩 | gorilla | 1 | 2 | 2.86 | 3.50 | 4.28 | 2.70 | 2.29 | 0.60 | 1.99 | 0.90 | 4.00 | 4.42 | 0.00 | 1025 |
| 葡萄 | grape | 1 | 2 | 3.14 | 4.56 | 4.75 | 2.89 | 3.17 | 0.98 | 0.17 | 0.98 | 1.81 | 2.86 | 0.00 | 806 |
| 草 | grass | 5 | 1 | 3.07 | 3.57 | 4.47 | 2.75 | 3.37 | 0.48 | 2.19 | 0.98 | 1.44 | 3.56 | 0.06 | 1072 |
| 蝗虫 | grasshopper | 1 | 2 | 2.64 | 4.45 | 4.44 | 4.05 | 1.80 | 0.33 | 2.32 | 0.80 | 4.88 | 10.67 | 0.09 | 1296 |
| 吉他 | guitar | 1 | 2 | 2.90 | 4.42 | 4.69 | 3.35 | 2.37 | 0.80 | 0.83 | 0.80 | 5.53 | 10.53 | 0.06 | 992 |
| 手枪 | gun | 1 | 2 | 3.19 | 4.08 | 4.39 | 2.95 | 2.23 | 0.73 | 1.15 | 1.00 | 3.81 | 2.74 | 0.00 | 716 |
| 头发 | hair | 1 | 2 | 2.76 | 2.74 | 3.36 | 2.81 | 4.26 | 0.55 | 1.81 | 0.63 | 2.06 | 10.40 | 0.23 | 1275 |
| 汉堡 | hamburger | 1 | 2 | 2.64 | 4.58 | 4.14 | 3.46 | 3.40 | 0.65 | 1.29 | 0.80 | 6.12 | 3.81 | 0.06 | 1222 |
| 锤子 | hammer | 1 | 2 | 3.12 | 4.11 | 4.72 | 2.22 | 2.31 | 0.58 | 2.23 | 0.80 | 3.06 | 4.66 | 0.00 | 1148 |
| 手 | hand | 1 | 1 | 3.40 | 4.42 | 4.92 | 2.83 | 4.40 | 0.73 | 1.21 | 1.00 | 1.24 | 2.25 | 0.03 | 726 |
| 手铐 | handcuff | 4 | 2 | 3.24 | 4.69 | 4.36 | 2.89 | 1.77 | 0.95 | 0.17 | 0.98 | 4.44 | 10.30 | 0.03 | 1001 |
| 衣架 | hanger | 1 | 2 | 2.55 | 4.17 | 4.97 | 1.16 | 3.89 | 0.83 | 1.04 | 0.98 | 3.44 | 3.74 | 0.06 | 980 |
| 竖琴 | harp | 1 | 2 | 3.14 | 4.13 | 3.36 | 3.24 | 1.39 | 0.40 | 1.46 | 0.40 | 5.56 | 10.30 | 0.14 | 1141 |
| 帽子 | hat | 1 | 2 | 3.31 | 3.51 | 4.47 | 2.14 | 3.46 | 0.65 | 1.44 | 0.98 | 2.31 | 4.84 | 0.03 | 688 |
| 耳机 | headphone | 1 | 2 | 2.76 | 3.61 | 4.53 | 3.81 | 4.00 | 0.63 | 1.21 | 0.95 | 5.69 | 10.36 | 0.09 | 1407 |
| 心 | heart | 1 | 1 | 2.76 | 4.09 | 4.83 | 1.16 | 3.69 | 0.63 | 1.79 | 1.00 | 2.81 | 4.58 | 0.03 | 856 |
| 刺猬 | hedgehog | 2 | 2 | 3.38 | 3.43 | 3.50 | 4.43 | 1.74 | 0.93 | 0.18 | 0.93 | 3.35 | 3.73 | 0.00 | 1033 |
| 直升机 | helicopter | 1 | 3 | 3.48 | 4.44 | 4.56 | 3.16 | 2.34 | 0.45 | 1.53 | 1.00 | 4.00 | 4.42 | 0.00 | 888 |
| 头盔 | helmet | 1 | 2 | 2.95 | 2.79 | 2.39 | 2.73 | 1.85 | 0.38 | 1.16 | 0.48 | 5.00 | 10.65 | 0.60 | 1809 |
| 母鸡 | hen | 1 | 2 | 2.24 | 4.36 | 4.92 | 2.86 | 2.51 | 0.83 | 0.67 | 1.00 | 2.24 | 10.40 | 0.03 | 836 |
| 啄木鸟 | woodpecker | 4 | 3 | 3.31 | 3.46 | 3.72 | 3.70 | 2.00 | 1.00 | 0.00 | 1.00 | 3.06 | 7.84 | 0.06 | 1209 |
| 河马 | hippo | 1 | 2 | 2.88 | 3.58 | 3.56 | 3.84 | 1.86 | 0.75 | 0.52 | 0.75 | 4.65 | 7.17 | 0.03 | 1223 |
| 马蹄 | hoof | 5 | 2 | 2.79 | 3.66 | 4.17 | 2.22 | 1.80 | 0.40 | 2.13 | 0.95 | 3.69 | 5.78 | 0.03 | 1218 |
| 钩子 | hook | 5 | 2 | 3.07 | 3.83 | 3.78 | 1.32 | 2.51 | 0.53 | 2.30 | 0.93 | 3.00 | 3.78 | 0.03 | 1069 |
| 圆号 | horn | 1 | 2 | 2.86 | 3.29 | 4.03 | 4.27 | 2.14 | 0.35 | 2.40 | 0.70 | 5.00 | 4.58 | 0.11 | 1315 |
| 马 | horse | 1 | 1 | 3.12 | 4.22 | 4.75 | 3.35 | 2.66 | 0.98 | 0.17 | 1.00 | 2.06 | 2.30 | 0.00 | 916 |
| 房子 | house | 1 | 2 | 3.86 | 3.83 | 4.69 | 3.59 | 3.91 | 0.68 | 1.79 | 1.00 | 1.69 | 2.30 | 0.00 | 831 |
| 熨斗 | iron | 1 | 2 | 2.55 | 4.53 | 4.25 | 3.03 | 1.94 | 0.85 | 0.73 | 0.98 | 3.88 | 10.59 | 0.00 | 995 |
| 外套 | jacket | 1 | 2 | 3.45 | 3.72 | 4.86 | 2.76 | 3.71 | 0.28 | 2.85 | 0.75 | 3.47 | 11.00 | 0.00 | 872 |
| 洗手池 | jar | 1 | 3 | 3.38 | 2.66 | 4.06 | 2.54 | 3.60 | 0.08 | 4.29 | 0.60 | 2.82 | 10.32 | 0.14 | 985 |
| 法官 | judge | 5 | 2 | 3.31 | 3.29 | 4.08 | 4.27 | 2.26 | 0.85 | 0.64 | 0.93 | 5.13 | 10.60 | 0.06 | 1063 |
| 袋鼠 | kangaroo | 1 | 2 | 2.98 | 4.43 | 4.50 | 3.22 | 1.85 | 0.95 | 0.34 | 0.98 | 4.38 | 3.73 | 0.00 | 892 |
| 水壶 | kettle | 1 | 2 | 3.05 | 3.09 | 4.75 | 2.54 | 3.80 | 0.80 | 1.17 | 1.00 | 2.69 | 3.78 | 0.06 | 868 |
| 钥匙 | key | 1 | 2 | 3.19 | 4.39 | 4.97 | 2.08 | 4.31 | 1.00 | 0.00 | 1.00 | 2.63 | 2.79 | 0.03 | 694 |
| 厨房 | kitch | 2 | 2 | 2.52 | 3.49 | 4.00 | 3.68 | 2.91 | 0.68 | 0.78 | 0.68 | 3.00 | 5.78 | 0.06 | 1283 |
| 风筝 | kite | 1 | 2 | 3.55 | 3.31 | 4.25 | 2.24 | 2.40 | 0.95 | 0.17 | 0.95 | 3.35 | 4.58 | 0.03 | 849 |
| 刀 | knife | 1 | 1 | 2.83 | 2.86 | 3.22 | 1.38 | 3.20 | 0.23 | 2.56 | 0.80 | 2.35 | 3.56 | 0.31 | 1095 |
| 梯子 | ladder | 1 | 2 | 3.24 | 4.18 | 4.69 | 2.11 | 2.59 | 0.78 | 1.09 | 0.98 | 2.47 | 3.78 | 0.03 | 888 |
| 羊 | lamb | 1 | 1 | 2.95 | 3.39 | 4.22 | 2.65 | 2.50 | 0.38 | 2.31 | 0.85 | 3.13 | 10.80 | 0.09 | 1278 |
| 台灯 | lamp | 1 | 2 | 2.93 | 3.00 | 4.58 | 1.70 | 3.89 | 0.90 | 0.30 | 0.95 | 3.53 | 4.74 | 0.00 | 941 |
| 灯笼 | lantern | 5 | 2 | 3.55 | 4.00 | 4.58 | 2.22 | 2.03 | 1.00 | 0.00 | 1.00 | 2.31 | 4.17 | 0.00 | 937 |
| 割草机 | lawnmower | 1 | 3 | 2.60 | 3.68 | 3.11 | 4.57 | 1.71 | 0.25 | 1.42 | 0.55 | 6.18 | 10.77 | 0.34 | 1703 |
| 树叶 | leaf | 1 | 2 | 3.05 | 3.64 | 4.72 | 2.32 | 3.54 | 0.43 | 1.67 | 1.00 | 2.07 | 2.30 | 0.00 | 886 |
| 腿 | leg | 1 | 1 | 3.60 | 3.97 | 4.97 | 2.00 | 4.09 | 0.73 | 1.60 | 1.00 | 1.56 | 3.41 | 0.00 | 904 |
| 柠檬 | lemon | 1 | 2 | 2.40 | 3.93 | 2.97 | 1.59 | 2.91 | 0.33 | 2.23 | 0.33 | 5.44 | 7.09 | 0.17 | 1434 |
| 豹子 | leopard | 5 | 2 | 2.65 | 3.82 | 4.14 | 4.35 | 1.77 | 0.48 | 1.66 | 0.93 | 3.94 | 4.69 | 0.09 | 1147 |
| 信 | letter | 3 | 1 | 3.10 | 3.36 | 4.34 | 3.92 | 3.11 | 0.60 | 1.84 | 0.93 | 3.59 | 10.68 | 0.00 | 1327 |
| 卷心菜 | lettuce | 1 | 3 | 3.02 | 2.91 | 2.86 | 3.19 | 3.00 | 0.20 | 2.80 | 0.55 | 4.31 | 10.87 | 0.23 | 1239 |
| 灯泡 | lightbulb | 1 | 2 | 2.81 | 4.33 | 4.89 | 2.76 | 3.14 | 0.90 | 0.62 | 1.00 | 2.19 | 3.41 | 0.00 | 729 |
| 狮子 | lion | 1 | 2 | 2.54 | 3.88 | 4.33 | 3.49 | 2.14 | 0.90 | 0.55 | 1.00 | 3.13 | 3.78 | 0.06 | 1012 |
| 蜥蜴 | lizard | 1 | 2 | 2.90 | 3.45 | 3.42 | 2.14 | 1.76 | 0.70 | 1.02 | 0.70 | 4.88 | 10.53 | 0.06 | 1410 |
| 虾 | lobster | 5 | 1 | 2.55 | 4.06 | 4.61 | 2.59 | 2.89 | 0.68 | 1.34 | 0.93 | 3.53 | 4.69 | 0.03 | 1247 |
| 锁 | lock | 1 | 1 | 2.74 | 4.46 | 4.97 | 2.11 | 3.53 | 0.90 | 0.47 | 1.00 | 2.63 | 5.68 | 0.00 | 855 |
| 藕 | lotus | 5 | 1 | 3.24 | 4.31 | 4.58 | 3.27 | 2.89 | 0.73 | 1.06 | 0.93 | 4.19 | 8.67 | 0.00 | 944 |
| 机关枪 | machine gun | 2 | 3 | 2.88 | 3.74 | 3.78 | 4.27 | 1.77 | 0.45 | 2.21 | 0.78 | 3.71 | 9.16 | 0.06 | 1160 |
| 放大镜 | magnifier | 2 | 3 | 2.88 | 4.72 | 4.47 | 2.19 | 2.37 | 0.98 | 0.17 | 0.98 | 3.94 | 7.17 | 0.06 | 1152 |
| 螳螂 | mantis | 5 | 2 | 2.74 | 4.10 | 4.39 | 3.95 | 1.86 | 0.65 | 0.95 | 0.65 | 4.13 | 10.27 | 0.06 | 1092 |
| 地图 | map | 3 | 2 | 3.05 | 3.57 | 4.28 | 4.24 | 3.26 | 0.88 | 0.68 | 0.95 | 3.35 | 7.80 | 0.03 | 1017 |
| 海军 | marine | 2 | 2 | 3.31 | 3.97 | 4.26 | 3.24 | 2.06 | 0.53 | 2.08 | 0.75 | 3.63 | 7.09 | 0.17 | 1052 |
| 火柴 | match | 4 | 2 | 2.50 | 3.80 | 4.69 | 1.92 | 2.26 | 0.90 | 0.51 | 0.95 | 2.44 | 10.47 | 0.00 | 960 |
| 西瓜 | melon | 1 | 2 | 2.48 | 2.86 | 4.56 | 2.22 | 2.91 | 0.88 | 0.47 | 0.88 | 2.35 | 2.79 | 0.00 | 972 |
| 美人鱼 | mermaid | 2 | 3 | 2.57 | 3.42 | 4.11 | 4.16 | 1.83 | 0.93 | 0.34 | 0.95 | 3.94 | 4.72 | 0.00 | 1186 |
| 话筒 | microphone | 2 | 2 | 2.67 | 4.14 | 4.83 | 3.11 | 3.17 | 0.48 | 1.42 | 0.98 | 4.82 | 5.74 | 0.00 | 963 |
| 显微镜 | microscope | 1 | 3 | 2.67 | 3.89 | 4.22 | 4.00 | 2.20 | 0.88 | 0.52 | 0.88 | 5.63 | 10.86 | 0.14 | 1431 |
| 镜子 | mirror | 4 | 2 | 3.14 | 3.56 | 4.44 | 2.54 | 3.94 | 0.78 | 0.95 | 0.93 | 1.81 | 3.18 | 0.03 | 994 |
| 手套 | mitten | 1 | 2 | 3.22 | 3.53 | 4.72 | 2.03 | 3.17 | 0.90 | 0.47 | 1.00 | 2.41 | 3.73 | 0.00 | 829 |
| 钞票 | money | 5 | 2 | 3.19 | 3.44 | 4.33 | 4.61 | 4.29 | 0.35 | 2.40 | 0.93 | 3.88 | 4.56 | 0.03 | 955 |
| 和尚 | monk | 5 | 2 | 2.93 | 3.47 | 4.08 | 3.19 | 1.94 | 0.43 | 3.02 | 0.78 | 3.44 | 5.73 | 0.06 | 1097 |
| 猴子 | monkey | 1 | 2 | 2.93 | 3.94 | 4.56 | 3.03 | 2.37 | 0.80 | 1.03 | 1.00 | 3.41 | 2.85 | 0.00 | 898 |
| 月亮 | moon | 1 | 2 | 2.95 | 4.11 | 4.75 | 1.03 | 3.49 | 0.63 | 1.69 | 1.00 | 1.81 | 2.69 | 0.00 | 743 |
| 牵牛花 | morning glory | 5 | 3 | 2.60 | 3.20 | 3.86 | 4.56 | 1.84 | 0.55 | 1.95 | 0.68 | 3.06 | 10.65 | 0.14 | 1521 |
| 蚊子 | mosquito | 5 | 2 | 3.10 | 3.50 | 4.22 | 2.68 | 2.71 | 0.60 | 1.56 | 0.63 | 2.00 | 10.33 | 0.06 | 1395 |
| 摩托车 | motorcycle | 1 | 3 | 3.02 | 4.03 | 4.81 | 4.46 | 2.83 | 0.70 | 0.88 | 1.00 | 4.50 | 3.67 | 0.00 | 867 |
| 山 | mountain | 1 | 1 | 3.45 | 3.23 | 4.25 | 2.19 | 3.40 | 0.40 | 2.31 | 1.00 | 1.94 | 3.42 | 0.00 | 972 |
| 老鼠 | mouse | 1 | 2 | 2.79 | 4.61 | 4.64 | 3.05 | 2.50 | 0.90 | 0.62 | 1.00 | 2.29 | 2.85 | 0.00 | 798 |
| 鼠标 | mouse | 4 | 2 | 2.93 | 3.75 | 4.78 | 2.62 | 4.31 | 0.98 | 0.00 | 0.98 | 6.56 | 6.94 | 0.00 | 1040 |
| 嘴唇 | mouth | 1 | 2 | 2.90 | 4.47 | 4.81 | 1.43 | 3.71 | 0.43 | 1.91 | 0.98 | 2.19 | 3.18 | 0.00 | 755 |
| 蘑菇 | mushroom | 1 | 2 | 3.19 | 4.33 | 4.58 | 2.86 | 3.14 | 0.98 | 0.17 | 1.00 | 3.41 | 2.79 | 0.00 | 834 |
| 胡子 | mustache | 3 | 2 | 2.81 | 2.97 | 3.56 | 3.76 | 3.15 | 0.50 | 1.67 | 0.58 | 2.69 | 8.92 | 0.14 | 1374 |
| 钉子 | nail | 1 | 2 | 2.90 | 4.60 | 4.58 | 1.46 | 2.66 | 0.75 | 1.12 | 0.90 | 2.63 | 7.44 | 0.03 | 877 |
| 项链 | necklace | 1 | 2 | 3.33 | 3.69 | 4.39 | 1.73 | 2.94 | 0.83 | 1.04 | 0.90 | 3.81 | 5.74 | 0.00 | 860 |
| 针 | needle | 1 | 1 | 3.32 | 4.39 | 4.22 | 1.49 | 2.74 | 0.80 | 0.93 | 0.88 | 2.44 | 7.44 | 0.06 | 933 |
| 鱼网 | net | 1 | 2 | 2.76 | 2.29 | 4.64 | 2.89 | 1.89 | 0.15 | 3.29 | 0.75 | 4.00 | 6.69 | 0.06 | 979 |
| 鼻子 | nose | 1 | 2 | 2.74 | 4.03 | 4.64 | 1.62 | 3.89 | 0.85 | 0.55 | 0.98 | 1.69 | 3.74 | 0.00 | 833 |
| 护士 | nurse | 2 | 2 | 3.14 | 3.06 | 4.47 | 4.24 | 2.60 | 0.75 | 1.25 | 0.80 | 3.29 | 10.59 | 0.00 | 929 |
| 螺丝帽 | nut | 1 | 3 | 2.55 | 4.12 | 4.69 | 2.14 | 2.17 | 0.25 | 2.95 | 0.73 | 4.50 | 10.41 | 0.06 | 1307 |
| 洋葱 | onion | 1 | 2 | 2.98 | 3.71 | 3.97 | 2.84 | 2.69 | 0.43 | 2.19 | 0.83 | 4.35 | 6.69 | 0.03 | 1294 |
| 桔子 | orange | 1 | 2 | 3.45 | 3.56 | 3.44 | 2.14 | 3.43 | 0.53 | 1.54 | 0.78 | 2.00 | 5.78 | 0.11 | 1330 |
| 鸵鸟 | ostrich | 1 | 2 | 2.95 | 4.09 | 4.08 | 2.89 | 1.89 | 0.88 | 0.63 | 0.88 | 4.50 | 10.27 | 0.09 | 1137 |
| 猫头鹰 | owl | 1 | 3 | 2.52 | 4.19 | 4.28 | 3.84 | 2.00 | 0.98 | 0.17 | 0.98 | 3.65 | 4.68 | 0.00 | 866 |
| 毛笔 | paintbrush | 1 | 2 | 3.55 | 3.64 | 4.11 | 1.92 | 2.23 | 0.85 | 0.78 | 0.90 | 3.00 | 4.73 | 0.00 | 1048 |
| 平底锅 | pan | 1 | 3 | 2.57 | 4.14 | 3.97 | 2.11 | 2.29 | 0.40 | 2.07 | 0.68 | 5.25 | 5.65 | 0.11 | 1449 |
| 熊猫 | panda | 5 | 2 | 2.71 | 4.64 | 4.78 | 3.62 | 2.56 | 0.88 | 0.63 | 1.00 | 2.71 | 3.41 | 0.00 | 704 |
| 裤子 | pants | 1 | 2 | 3.60 | 4.33 | 4.78 | 1.92 | 4.34 | 0.93 | 0.50 | 1.00 | 1.94 | 4.20 | 0.00 | 772 |
| 礼物 | parcel | 4 | 2 | 3.33 | 4.03 | 4.28 | 2.38 | 3.23 | 0.45 | 2.33 | 0.93 | 3.56 | 4.56 | 0.00 | 1146 |
| 鹦鹉 | parrot | 1 | 2 | 3.36 | 4.00 | 4.11 | 3.46 | 2.14 | 0.63 | 1.62 | 0.63 | 4.19 | 7.86 | 0.09 | 1083 |
| 桃子 | peach | 1 | 2 | 2.95 | 3.83 | 3.61 | 1.65 | 2.74 | 0.40 | 2.26 | 0.60 | 2.00 | 7.93 | 0.20 | 1420 |
| 孔雀 | peacock | 1 | 2 | 2.81 | 4.03 | 4.56 | 3.92 | 2.00 | 0.95 | 0.34 | 1.00 | 3.13 | 5.74 | 0.00 | 825 |
| 花生 | peanut | 1 | 2 | 2.48 | 4.36 | 4.33 | 2.59 | 3.29 | 0.95 | 0.00 | 0.95 | 2.06 | 4.42 | 0.09 | 1100 |
| 梨 | pear | 1 | 1 | 2.81 | 4.08 | 4.58 | 1.16 | 3.23 | 0.60 | 1.66 | 0.70 | 1.71 | 2.69 | 0.00 | 1109 |
| 圆珠笔 | pen | 1 | 3 | 2.98 | 3.53 | 4.81 | 2.22 | 4.20 | 0.35 | 1.58 | 1.00 | 3.56 | 10.27 | 0.03 | 891 |
| 铅笔 | pencil | 1 | 2 | 3.36 | 4.36 | 4.80 | 2.22 | 3.51 | 0.95 | 0.29 | 1.00 | 2.06 | 2.85 | 0.00 | 843 |
| 企鹅 | penguin | 1 | 2 | 3.17 | 3.75 | 3.81 | 2.16 | 2.23 | 0.95 | 0.00 | 0.95 | 3.81 | 3.58 | 0.03 | 960 |
| 青椒 | pepper | 1 | 2 | 3.05 | 4.03 | 4.44 | 2.11 | 3.37 | 0.28 | 2.75 | 0.73 | 3.88 | 10.70 | 0.11 | 1245 |
| 钢琴 | piano | 1 | 2 | 3.02 | 3.94 | 4.47 | 4.11 | 2.54 | 0.85 | 0.64 | 0.98 | 3.63 | 4.20 | 0.00 | 936 |
| 猪 | pig | 1 | 1 | 3.05 | 4.03 | 4.78 | 2.39 | 3.26 | 0.85 | 0.88 | 1.00 | 1.63 | 3.68 | 0.03 | 927 |
| 鸽子 | pigeon | 2 | 2 | 3.21 | 3.97 | 4.47 | 3.38 | 2.44 | 0.38 | 2.26 | 0.43 | 2.44 | 10.40 | 0.09 | 905 |
| 存钱罐 | piggybank | 2 | 3 | 2.71 | 3.81 | 4.50 | 2.81 | 2.14 | 0.35 | 2.76 | 0.90 | 3.82 | 7.89 | 0.09 | 1071 |
| 枕头 | pillow | 2 | 2 | 2.76 | 3.56 | 3.92 | 2.81 | 4.09 | 0.73 | 0.78 | 0.73 | 1.81 | 3.73 | 0.03 | 1122 |
| 图钉 | pin | 2 | 2 | 3.40 | 4.47 | 3.83 | 1.92 | 2.37 | 0.70 | 1.37 | 0.93 | 4.00 | 10.86 | 0.11 | 1160 |
| 菠萝 | pineapple | 1 | 2 | 3.17 | 4.53 | 4.81 | 3.92 | 3.29 | 0.93 | 0.45 | 0.98 | 3.33 | 3.78 | 0.03 | 807 |
| 烟斗 | pipe | 1 | 2 | 3.07 | 4.14 | 4.39 | 1.70 | 1.89 | 0.95 | 0.34 | 0.98 | 4.00 | 10.45 | 0.00 | 917 |
| 海盗 | pirate | 3 | 2 | 3.31 | 3.91 | 3.89 | 4.59 | 1.86 | 0.58 | 2.58 | 0.85 | 5.00 | 10.50 | 0.00 | 1483 |
| 水壶 | pitcher | 1 | 2 | 3.48 | 2.24 | 4.42 | 1.84 | 3.91 | 0.23 | 3.00 | 0.75 | #NULL! | #NULL! | 0.00 | 1143 |
| 行星 | planet | 5 | 2 | 2.24 | 3.58 | 3.67 | 2.22 | 1.88 | 0.15 | 3.31 | 0.78 | 5.13 | 7.89 | 0.23 | 1388 |
| 盘子 | plate | 5 | 2 | 2.83 | 4.44 | 3.91 | 1.62 | 3.63 | 0.40 | 2.43 | 0.65 | 2.53 | 7.71 | 0.17 | 1155 |
| 钳子 | pliers | 1 | 2 | 3.10 | 3.66 | 4.50 | 2.16 | 2.17 | 0.65 | 1.64 | 0.85 | 3.82 | 8.92 | 0.06 | 1158 |
| 插头 | plug | 1 | 2 | 3.14 | 4.19 | 4.78 | 2.00 | 3.71 | 0.83 | 0.84 | 0.93 | 3.69 | 10.33 | 0.00 | 1105 |
| 梅花 | plum blossom | 5 | 2 | 2.45 | 4.17 | 4.56 | 3.00 | 2.12 | 0.93 | 0.50 | 0.98 | 3.41 | 10.55 | 0.00 | 923 |
| 警察 | policeman | 5 | 2 | 2.74 | 4.06 | 4.58 | 4.42 | 3.14 | 0.80 | 1.13 | 0.95 | 2.88 | 2.69 | 0.03 | 830 |
| 土豆 | potato | 1 | 2 | 2.57 | 3.61 | 3.56 | 1.30 | 4.09 | 0.70 | 0.82 | 0.78 | 2.81 | 4.66 | 0.06 | 1220 |
| 婴儿车 | pram | 1 | 3 | 2.33 | 3.56 | 3.89 | 3.24 | 1.91 | 0.55 | 1.66 | 0.70 | 4.50 | 10.35 | 0.14 | 1227 |
| 幻灯机 | project | 2 | 3 | 3.21 | 3.91 | 3.06 | 3.41 | 2.82 | 0.23 | 3.10 | 0.45 | 5.69 | 10.95 | 0.29 | 1677 |
| 南瓜 | pumpkin | 1 | 2 | 2.55 | 4.06 | 4.42 | 2.24 | 2.46 | 0.93 | 0.34 | 0.95 | 3.50 | 5.72 | 0.03 | 908 |
| 金字塔 | pyramid | 3 | 3 | 2.93 | 4.39 | 4.03 | 3.76 | 1.91 | 0.75 | 1.25 | 0.75 | 5.24 | 10.27 | 0.00 | 1089 |
| 兔子 | rabbit | 1 | 2 | 3.48 | 4.29 | 4.89 | 2.73 | 2.74 | 0.68 | 1.41 | 1.00 | 2.25 | 1.94 | 0.00 | 716 |
| 收音机 | radio | 4 | 3 | 2.45 | 3.83 | 4.50 | 4.05 | 3.43 | 0.80 | 0.89 | 0.98 | 3.25 | 4.56 | 0.00 | 990 |
| 萝卜 | radish | 5 | 2 | 3.50 | 3.39 | 4.53 | 3.51 | 3.17 | 0.85 | 0.95 | 0.95 | 2.31 | 3.73 | 0.00 | 930 |
| 竹排 | raft | 2 | 2 | 3.10 | 4.28 | 3.56 | 3.97 | 1.77 | 0.25 | 2.52 | 0.73 | 5.06 | 10.40 | 0.20 | 1266 |
| 铁轨 | rail | 5 | 2 | 2.81 | 4.22 | 4.44 | 3.35 | 2.40 | 0.53 | 2.14 | 0.93 | 4.47 | 7.80 | 0.06 | 1272 |
| 唱片机 | record player | 1 | 3 | 2.95 | 3.53 | 3.51 | 3.53 | 2.09 | 0.25 | 2.34 | 0.50 | 5.24 | 10.95 | 0.17 | 1523 |
| 冰箱 | refrigerator | 1 | 2 | 2.93 | 4.11 | 4.53 | 2.03 | 2.94 | 0.88 | 0.63 | 0.93 | 4.59 | 3.58 | 0.00 | 857 |
| 犀牛 | rhinoceros | 1 | 2 | 2.40 | 4.26 | 4.03 | 3.51 | 1.63 | 0.78 | 0.85 | 0.78 | 4.82 | 5.78 | 0.06 | 1046 |
| 戒指 | ring | 1 | 2 | 3.57 | 3.94 | 3.89 | 2.14 | 2.74 | 0.63 | 1.31 | 0.63 | 4.50 | 10.60 | 0.09 | 1310 |
| 公路 | road | 5 | 2 | 3.21 | 3.36 | 4.47 | 3.73 | 3.50 | 0.73 | 1.26 | 0.98 | 3.06 | 7.17 | 0.03 | 1248 |
| 机器人 | robot | 2 | 3 | 2.60 | 3.75 | 4.31 | 3.78 | 2.09 | 1.00 | 0.00 | 1.00 | 4.31 | 3.41 | 0.00 | 808 |
| 火箭 | rocket | 1 | 2 | 3.10 | 4.11 | 4.33 | 3.92 | 2.26 | 0.95 | 0.17 | 0.98 | 4.00 | 4.72 | 0.06 | 1007 |
| 椅子 | rocking chair | 1 | 2 | 3.29 | 3.14 | 4.22 | 3.39 | 4.24 | 0.88 | 1.91 | 0.98 | 4.81 | 4.66 | 0.06 | 855 |
| 擀面杖 | rolling pin | 1 | 3 | 2.81 | 3.42 | 3.22 | 1.38 | 1.83 | 0.13 | 2.64 | 0.18 | 3.38 | 10.60 | 0.31 | 1479 |
| 屋顶 | roof | 4 | 2 | 2.93 | 3.97 | 4.83 | 1.73 | 3.03 | 0.43 | 2.27 | 0.73 | 3.25 | 7.84 | 0.00 | 844 |
| 公鸡 | rooster | 1 | 2 | 2.76 | 4.56 | 4.83 | 3.27 | 2.23 | 0.85 | 0.80 | 0.98 | 2.06 | 2.69 | 0.00 | 760 |
| 绳子 | rope | 1 | 2 | 3.79 | 4.39 | 4.72 | 3.35 | 3.00 | 0.53 | 2.36 | 0.95 | 2.06 | 5.16 | 0.00 | 892 |
| 玫瑰 | rose | 2 | 2 | 3.36 | 4.00 | 4.56 | 3.62 | 3.14 | 0.63 | 1.35 | 0.93 | 5.19 | 7.00 | 0.00 | 867 |
| 地毯 | rug | 5 | 2 | 3.00 | 3.08 | 4.23 | 3.38 | 2.71 | 0.35 | 2.76 | 0.63 | 4.38 | 10.77 | 0.03 | 1026 |
| 尺子 | rule | 1 | 2 | 3.40 | 4.81 | 4.86 | 2.11 | 3.06 | 0.65 | 1.32 | 1.00 | 2.69 | 4.66 | 0.00 | 791 |
| 保险柜 | safe | 4 | 3 | 2.95 | 4.08 | 4.17 | 2.46 | 2.11 | 0.38 | 2.60 | 0.53 | 5.56 | 10.35 | 0.06 | 1167 |
| 帆船 | sailboat | 1 | 2 | 2.38 | 4.11 | 4.39 | 3.00 | 2.00 | 0.83 | 0.77 | 0.98 | 3.76 | 7.89 | 0.00 | 920 |
| 凉鞋 | sandals | 4 | 2 | 3.48 | 3.03 | 4.72 | 3.14 | 3.18 | 0.73 | 1.34 | 0.93 | 2.38 | 10.60 | 0.00 | 1010 |
| 三明治 | sandwich | 1 | 3 | 2.95 | 3.00 | 3.61 | 3.00 | 2.83 | 0.45 | 2.52 | 0.78 | 5.88 | 9.03 | 0.17 | 1461 |
| 水瓢 | saucepan | 1 | 2 | 2.59 | 3.53 | 4.33 | 1.84 | 1.66 | 0.13 | 3.82 | 0.63 | 5.56 | 10.64 | 0.14 | 1427 |
| 香肠 | sausage | 5 | 2 | 3.55 | 3.36 | 3.53 | 1.46 | 3.12 | 0.33 | 2.51 | 0.45 | 3.88 | 7.09 | 0.23 | 1704 |
| 锯 | saw | 1 | 1 | 2.48 | 3.23 | 3.06 | 2.11 | 1.80 | 0.28 | 2.19 | 0.63 | 3.44 | 9.16 | 0.14 | 1240 |
| 天平 | scale | 1 | 2 | 2.67 | 3.78 | 4.47 | 2.81 | 2.23 | 0.88 | 0.63 | 0.98 | 4.53 | 10.47 | 0.00 | 979 |
| 围巾 | scarf | 3 | 2 | 2.81 | 4.39 | 4.78 | 3.32 | 2.80 | 1.00 | 0.00 | 1.00 | 2.94 | 3.41 | 0.03 | 1009 |
| 剪刀 | scissors | 1 | 2 | 3.05 | 4.11 | 4.78 | 2.05 | 3.40 | 0.88 | 0.54 | 1.00 | 2.19 | 1.94 | 0.03 | 726 |
| 乐谱 | score | 2 | 2 | 3.24 | 3.86 | 4.40 | 4.46 | 1.97 | 0.45 | 1.71 | 1.00 | 4.94 | 7.89 | 0.03 | 1127 |
| 蝎子 | scorpion | 1 | 2 | 2.55 | 4.12 | 3.92 | 3.54 | 1.68 | 0.68 | 1.32 | 0.70 | 4.35 | 10.50 | 0.14 | 1204 |
| 螺丝钉 | screw | 1 | 3 | 2.19 | 4.31 | 4.11 | 2.86 | 2.29 | 0.35 | 2.59 | 0.70 | 3.88 | 10.32 | 0.09 | 1163 |
| 螺丝刀 | screwdriver | 1 | 3 | 2.83 | 3.73 | 4.75 | 2.11 | 2.17 | 0.48 | 1.76 | 0.83 | 4.63 | 7.44 | 0.09 | 1355 |
| 海豹 | seal | 3 | 2 | 2.31 | 3.94 | 3.61 | 3.49 | 1.91 | 0.45 | 1.84 | 0.53 | 5.00 | 10.35 | 0.03 | 1356 |
| 葱 | scallion | 5 | 1 | 2.88 | 4.31 | 4.58 | 2.86 | 3.26 | 0.73 | 1.20 | 0.98 | 2.38 | 6.69 | 0.09 | 1066 |
| 鲨鱼 | shark | 5 | 2 | 2.50 | 4.17 | 3.89 | 3.73 | 1.89 | 0.70 | 1.35 | 0.73 | 4.56 | 3.56 | 0.06 | 1000 |
| 绵羊 | sheep | 1 | 2 | 2.98 | 3.56 | 4.17 | 3.05 | 2.03 | 0.48 | 1.73 | 0.83 | 2.81 | 10.40 | 0.03 | 1183 |
| 盾牌 | shield | 5 | 2 | 3.33 | 3.83 | 3.00 | 2.70 | 1.71 | 0.30 | 1.51 | 0.58 | 5.00 | 10.27 | 0.06 | 1216 |
| 衬衫 | shirt | 1 | 2 | 2.33 | 3.81 | 4.78 | 2.62 | 3.54 | 0.35 | 2.25 | 0.95 | 3.06 | 10.68 | 0.00 | 912 |
| 鞋 | shoe | 1 | 1 | 2.98 | 3.69 | 4.86 | 3.08 | 4.26 | 0.43 | 1.80 | 0.98 | 1.46 | 1.94 | 0.00 | 744 |
| 铲子 | shovel | 5 | 2 | 3.07 | 4.20 | 4.50 | 1.68 | 2.06 | 0.40 | 1.90 | 0.95 | 3.31 | 4.74 | 0.06 | 1063 |
| 蚕 | silkworm | 5 | 1 | 2.88 | 3.29 | 3.56 | 3.57 | 1.83 | 0.45 | 1.67 | 0.45 | 3.59 | 10.80 | 0.06 | 1125 |
| 溜冰鞋 | skate | 4 | 3 | 3.45 | 3.69 | 4.39 | 2.60 | 2.89 | 0.25 | 2.76 | 0.85 | 5.81 | 5.72 | 0.03 | 1166 |
| 裙子 | skirt | 1 | 2 | 3.05 | 3.11 | 4.31 | 1.32 | 3.12 | 0.65 | 1.55 | 0.95 | 2.44 | 4.18 | 0.03 | 956 |
| 骷髅 | skull | 1 | 2 | 2.81 | 4.25 | 4.25 | 3.70 | 1.71 | 0.60 | 1.62 | 1.00 | 5.69 | 9.03 | 0.00 | 910 |
| 雪橇 | sleigh | 1 | 2 | 2.74 | 3.00 | 2.86 | 3.00 | 1.80 | 0.35 | 2.00 | 0.50 | 5.56 | 10.45 | 0.26 | 1396 |
| 拖鞋 | slippers | 3 | 2 | 2.58 | 3.75 | 4.81 | 1.57 | 4.18 | 0.98 | 0.17 | 1.00 | 2.88 | 2.30 | 0.00 | 761 |
| 蜗牛 | snail | 1 | 2 | 2.45 | 4.47 | 4.56 | 2.68 | 1.94 | 0.98 | 0.00 | 0.98 | 3.69 | 3.56 | 0.00 | 913 |
| 蛇 | snake | 1 | 1 | 3.12 | 4.25 | 4.58 | 3.76 | 2.29 | 0.93 | 0.29 | 0.98 | 2.56 | 3.18 | 0.03 | 783 |
| 雪花 | snow | 5 | 2 | 3.55 | 3.89 | 4.31 | 2.73 | 2.37 | 0.90 | 0.35 | 0.93 | 2.31 | 4.74 | 0.00 | 1140 |
| 雪人 | snowman | 1 | 2 | 2.76 | 4.26 | 4.75 | 2.24 | 1.89 | 0.98 | 0.17 | 1.00 | 2.50 | 2.79 | 0.00 | 896 |
| 足球 | soccer | 5 | 2 | 3.05 | 4.56 | 4.83 | 3.70 | 3.63 | 1.00 | 0.00 | 1.00 | 4.06 | 4.68 | 0.00 | 803 |
| 袜子 | socks | 1 | 2 | 3.19 | 4.33 | 4.81 | 1.97 | 4.29 | 0.88 | 0.78 | 0.98 | 1.88 | 2.54 | 0.00 | 803 |
| 扳手 | spanner | 4 | 2 | 2.86 | 4.52 | 4.75 | 3.38 | 1.91 | 0.30 | 1.70 | 0.60 | 5.00 | 10.23 | 0.00 | 1153 |
| 矛 | spear | 5 | 1 | 2.76 | 3.58 | 4.03 | 2.41 | 1.80 | 0.28 | 2.63 | 0.90 | 4.69 | 10.75 | 0.09 | 1296 |
| 地球仪 | tellurion | 4 | 3 | 2.93 | 4.11 | 4.72 | 3.05 | 2.49 | 1.00 | 0.00 | 1.00 | 4.13 | 7.17 | 0.00 | 841 |
| 蜘蛛 | spider | 1 | 2 | 2.40 | 3.75 | 4.22 | 2.78 | 2.37 | 0.83 | 0.00 | 0.83 | 2.76 | 5.18 | 0.11 | 1194 |
| 纺车 | spinning wheel | 1 | 2 | 2.78 | 3.22 | 2.94 | 3.59 | 1.63 | 0.45 | 2.18 | 0.65 | 5.13 | 10.86 | 0.31 | 1398 |
| 勺子 | spoon | 1 | 2 | 2.95 | 4.31 | 4.92 | 1.35 | 3.77 | 0.63 | 1.91 | 1.00 | 2.00 | 2.54 | 0.00 | 789 |
| 三角板 | square | 2 | 3 | 3.07 | 4.20 | 4.72 | 1.51 | 2.20 | 0.53 | 1.43 | 1.00 | 3.63 | 5.18 | 0.00 | 892 |
| 松鼠 | squirrel | 1 | 2 | 2.74 | 4.50 | 4.33 | 2.65 | 1.83 | 0.98 | 0.17 | 1.00 | 3.47 | 4.56 | 0.00 | 1075 |
| 台阶 | staircase | 5 | 2 | 3.05 | 4.33 | 4.81 | 1.83 | 3.54 | 0.45 | 2.03 | 1.00 | 3.53 | 3.67 | 0.00 | 869 |
| 印章 | seal | 2 | 2 | 3.62 | 3.83 | 4.61 | 1.86 | 2.40 | 0.70 | 1.39 | 0.93 | 4.88 | 6.69 | 0.03 | 1124 |
| 邮票 | stamp | 5 | 2 | 3.67 | 3.75 | 4.17 | 4.54 | 2.83 | 0.95 | 0.29 | 0.95 | 3.56 | 9.16 | 0.06 | 1071 |
| 五角星 | star | 1 | 3 | 2.95 | 4.61 | 4.97 | 1.05 | 2.63 | 0.68 | 1.11 | 1.00 | 2.31 | 2.54 | 0.00 | 753 |
| 方向盘 | steering wheel | 2 | 3 | 2.93 | 4.56 | 4.75 | 2.49 | 2.51 | 0.95 | 0.17 | 0.95 | 4.65 | 4.73 | 0.14 | 1338 |
| 听诊器 | stethoscope | 1 | 3 | 2.57 | 3.91 | 4.31 | 2.59 | 1.89 | 0.80 | 0.56 | 0.88 | 4.24 | 10.73 | 0.06 | 1284 |
| 凳子 | stool | 1 | 2 | 3.00 | 3.58 | 4.64 | 2.08 | 3.86 | 0.65 | 1.90 | 0.93 | 1.94 | 3.56 | 0.00 | 800 |
| 草莓 | strawberry | 1 | 2 | 2.83 | 4.28 | 4.53 | 2.62 | 3.09 | 1.00 | 0.00 | 1.00 | 3.18 | 2.69 | 0.00 | 1068 |
| 皮箱 | suitcase | 1 | 2 | 3.12 | 3.53 | 4.42 | 2.86 | 2.97 | 0.23 | 3.15 | 1.00 | 3.88 | 7.17 | 0.00 | 972 |
| 相扑 | sumo | 2 | 2 | 2.22 | 3.44 | 4.00 | 3.43 | 1.57 | 0.53 | 2.39 | 0.78 | 5.88 | 10.85 | 0.09 | 1153 |
| 太阳 | sun | 1 | 2 | 2.90 | 4.56 | 4.89 | 1.35 | 4.03 | 0.98 | 0.17 | 1.00 | 1.65 | 2.54 | 0.00 | 685 |
| 向日葵 | sunflower | 4 | 3 | 2.52 | 4.11 | 4.58 | 3.16 | 2.31 | 0.90 | 0.67 | 0.95 | 3.31 | 10.27 | 0.03 | 867 |
| 燕子 | swallow | 4 | 2 | 2.95 | 4.63 | 4.61 | 3.51 | 2.43 | 0.93 | 0.45 | 1.00 | 2.41 | 4.56 | 0.00 | 991 |
| 鹅 | swan | 1 | 1 | 3.20 | 3.75 | 4.58 | 2.41 | 2.21 | 0.65 | 1.14 | 0.65 | 3.63 | 9.07 | 0.00 | 1004 |
| 毛衣 | sweater | 1 | 2 | 3.52 | 3.83 | 4.89 | 2.78 | 3.23 | 0.70 | 1.66 | 0.78 | 2.94 | 8.64 | 0.00 | 904 |
| 秋千 | swing | 1 | 2 | 2.98 | 4.42 | 4.44 | 1.78 | 2.37 | 0.80 | 0.38 | 0.83 | 2.94 | 10.40 | 0.06 | 1251 |
| 剑 | sword | 5 | 1 | 3.38 | 3.84 | 4.39 | 1.76 | 2.06 | 0.95 | 0.34 | 0.98 | 3.63 | 4.73 | 0.06 | 1042 |
| 注射器 | syringe | 1 | 3 | 3.07 | 4.64 | 4.67 | 3.22 | 2.03 | 0.40 | 2.08 | 1.00 | 4.13 | 4.72 | 0.00 | 942 |
| 乒乓球拍 | table tennis bat | 1 | 4 | 2.85 | 4.50 | 4.92 | 1.97 | 2.69 | 0.75 | 1.14 | 1.00 | 4.47 | 5.65 | 0.03 | 973 |
| 桌子 | table | 1 | 2 | 3.02 | 4.14 | 4.67 | 2.19 | 4.23 | 0.70 | 1.50 | 0.95 | 2.00 | 2.85 | 0.00 | 906 |
| 尾巴 | tail | 5 | 2 | 3.29 | 2.94 | 3.61 | 2.46 | 2.56 | 0.58 | 1.85 | 0.90 | 2.63 | 5.19 | 0.20 | 1340 |
| 坦克 | tank | 2 | 2 | 3.43 | 3.92 | 4.31 | 2.35 | 2.00 | 0.93 | 0.18 | 0.93 | 3.88 | 4.69 | 0.03 | 1012 |
| 靶子 | target | 2 | 2 | 3.10 | 3.40 | 4.25 | 3.46 | 1.83 | 0.20 | 3.29 | 0.75 | 4.24 | 10.47 | 0.26 | 1349 |
| 电话 | telephone | 1 | 2 | 3.00 | 3.50 | 4.78 | 3.32 | 4.46 | 0.88 | 0.63 | 1.00 | 4.19 | 2.54 | 0.03 | 752 |
| 望远镜 | telescope | 5 | 3 | 3.33 | 2.61 | 4.22 | 1.97 | 2.26 | 0.90 | 1.17 | 0.90 | 4.13 | 5.78 | 0.06 | 1153 |
| 电视 | television | 1 | 2 | 3.57 | 3.94 | 5.00 | 2.81 | 3.97 | 0.55 | 1.27 | 0.98 | 2.88 | 4.18 | 0.00 | 782 |
| 网球拍 | tennis racket | 1 | 4 | 2.90 | 4.22 | 4.61 | 3.30 | 2.88 | 0.43 | 2.03 | 0.45 | 4.31 | 11.00 | 0.00 | 877 |
| 帐篷 | tent | 3 | 2 | 2.69 | 4.19 | 4.47 | 2.27 | 2.11 | 0.93 | 0.34 | 0.93 | 4.63 | 5.74 | 0.00 | 963 |
| 试管 | test-tube | 2 | 2 | 2.83 | 3.42 | 3.47 | 1.86 | 2.26 | 0.78 | 0.90 | 0.85 | 5.75 | 11.00 | 0.31 | 1524 |
| 温度计 | thermograph | 1 | 3 | 2.81 | 3.58 | 4.64 | 2.95 | 2.69 | 0.93 | 0.50 | 0.98 | 4.06 | 7.80 | 0.00 | 1046 |
| 线轴 | thread spool | 1 | 2 | 2.95 | 3.32 | 4.11 | 3.32 | 1.71 | 0.30 | 3.04 | 0.68 | 4.75 | 7.84 | 0.09 | 1391 |
| 拇指 | thumb | 1 | 2 | 2.36 | 4.22 | 4.97 | 2.08 | 3.71 | 0.55 | 1.28 | 0.95 | 2.41 | 3.78 | 0.03 | 889 |
| 领带 | tie | 1 | 2 | 2.98 | 4.50 | 4.58 | 2.62 | 2.86 | 1.00 | 0.00 | 1.00 | 5.75 | 7.17 | 0.00 | 841 |
| 老虎 | tiger | 1 | 2 | 3.02 | 3.83 | 4.33 | 4.11 | 2.26 | 0.73 | 1.31 | 0.90 | 2.29 | 2.69 | 0.00 | 1009 |
| 轮胎 | tire | 1 | 2 | 3.62 | 4.33 | 4.67 | 3.57 | 2.80 | 0.65 | 1.47 | 0.98 | 3.73 | 6.94 | 0.00 | 912 |
| 马桶 | toilet | 2 | 2 | 2.26 | 4.19 | 4.72 | 2.22 | 3.40 | 0.73 | 1.35 | 1.00 | 4.88 | 9.19 | 0.03 | 995 |
| 西红柿 | tomato | 1 | 3 | 2.78 | 4.39 | 4.61 | 1.92 | 3.80 | 0.60 | 1.27 | 0.65 | 2.94 | 5.77 | 0.03 | 1186 |
| 舌头 | tongue | 5 | 2 | 2.64 | 3.14 | 4.64 | 2.05 | 3.54 | 0.70 | 1.26 | 0.85 | 1.94 | 3.73 | 0.03 | 1056 |
| 牙齿 | tooth | 5 | 2 | 3.64 | 2.28 | 4.17 | 2.32 | 4.00 | 0.38 | 2.25 | 0.75 | 1.81 | 4.73 | 0.03 | 1183 |
| 牙刷 | toothbrush | 1 | 2 | 2.98 | 4.39 | 4.92 | 2.62 | 4.43 | 0.98 | 0.00 | 1.00 | 2.25 | 3.42 | 0.00 | 773 |
| 陀螺 | top | 1 | 2 | 2.50 | 3.47 | 3.89 | 2.59 | 1.57 | 0.65 | 0.73 | 0.80 | 3.69 | 7.69 | 0.09 | 1194 |
| 手电筒 | torch | 5 | 3 | 2.81 | 4.03 | 4.83 | 2.73 | 2.57 | 0.65 | 1.49 | 0.95 | 3.06 | 7.92 | 0.00 | 1013 |
| 塔 | tower | 5 | 1 | 3.54 | 2.76 | 3.64 | 4.41 | 2.29 | 0.40 | 1.75 | 0.68 | 5.06 | 10.35 | 0.23 | 1255 |
| 毛巾 | towel | 5 | 2 | 3.19 | 4.22 | 4.36 | 2.30 | 4.34 | 0.90 | 0.35 | 0.95 | 2.19 | 4.42 | 0.03 | 994 |
| 拖拉机 | tractor | 1 | 3 | 3.26 | 3.54 | 4.03 | 4.19 | 2.00 | 0.55 | 2.04 | 0.65 | 3.06 | 10.55 | 0.11 | 1132 |
| 红绿灯 | traffic light | 1 | 3 | 2.67 | 3.92 | 4.44 | 2.92 | 3.83 | 0.48 | 1.30 | 0.85 | 3.75 | 5.68 | 0.06 | 1188 |
| 火车 | train | 1 | 2 | 3.10 | 4.08 | 4.81 | 4.32 | 3.17 | 0.98 | 0.17 | 1.00 | 3.56 | 2.30 | 0.03 | 861 |
| 大树 | tree | 1 | 2 | 3.86 | 4.31 | 4.81 | 3.46 | 3.83 | 0.50 | 1.29 | 0.98 | 2.00 | 2.30 | 0.00 | 856 |
| 树桩 | tree stump | 2 | 2 | 3.25 | 4.33 | 4.57 | 2.27 | 2.03 | 0.60 | 1.63 | 0.80 | 3.38 | 10.85 | 0.00 | 1122 |
| 三轮车 | tricycle | 4 | 3 | 2.57 | 2.22 | 4.06 | 3.78 | 2.50 | 0.20 | 3.14 | 0.40 | 3.44 | 10.86 | 0.11 | 1204 |
| 卡车 | truck | 1 | 2 | 2.88 | 3.56 | 4.64 | 2.54 | 2.71 | 0.38 | 2.24 | 0.88 | 2.93 | 7.09 | 0.03 | 1049 |
| 小号 | trumpet | 1 | 2 | 2.24 | 4.24 | 4.44 | 3.35 | 1.80 | 0.50 | 1.94 | 0.95 | 5.53 | 5.42 | 0.06 | 1082 |
| 海螺 | trumpet shell | 5 | 2 | 2.52 | 3.62 | 3.28 | 3.65 | 1.94 | 0.75 | 1.25 | 0.75 | 5.00 | 10.27 | 0.14 | 1395 |
| 郁金香 | tulip | 4 | 3 | 3.21 | 3.50 | 4.47 | 2.70 | 2.31 | 0.38 | 2.38 | 0.38 | 5.35 | 11.00 | 0.17 | 984 |
| 隧道 | tunnel | 2 | 2 | 3.05 | 3.97 | 4.03 | 3.89 | 2.14 | 0.50 | 1.87 | 0.70 | 5.35 | 10.70 | 0.11 | 1429 |
| 乌龟 | turtel | 1 | 2 | 2.83 | 4.46 | 4.47 | 3.16 | 2.40 | 0.80 | 0.72 | 1.00 | 3.00 | 2.54 | 0.00 | 754 |
| 雨伞 | umbrella | 1 | 2 | 3.71 | 4.47 | 4.89 | 2.68 | 3.03 | 0.58 | 1.21 | 1.00 | 2.76 | 1.94 | 0.00 | 698 |
| 花瓶 | vase | 1 | 2 | 3.05 | 3.94 | 4.42 | 3.16 | 2.97 | 0.80 | 0.98 | 0.95 | 3.00 | 4.42 | 0.00 | 913 |
| 小提琴 | violin | 1 | 3 | 2.43 | 4.42 | 4.42 | 3.89 | 2.03 | 0.48 | 2.28 | 0.65 | 4.50 | 5.74 | 0.09 | 906 |
| 排球 | volleyball | 5 | 2 | 2.74 | 4.22 | 4.61 | 2.03 | 2.66 | 0.70 | 1.42 | 0.70 | 4.63 | 10.27 | 0.06 | 968 |
| 马甲 | vest | 1 | 2 | 2.86 | 3.94 | 4.72 | 2.32 | 2.37 | 0.70 | 1.42 | 1.00 | 4.53 | 10.75 | 0.03 | 1034 |
| 背心 | waterfall | 4 | 2 | 3.24 | 4.36 | 4.83 | 1.97 | 3.34 | 0.88 | 0.68 | 0.90 | 2.40 | 4.42 | 0.03 | 935 |
| 墙 | wall | 5 | 1 | 3.69 | 3.56 | 4.86 | 3.81 | 3.37 | 0.68 | 1.48 | 0.90 | 2.25 | 3.81 | 0.00 | 1031 |
| 钱包 | wallet | 2 | 2 | 3.29 | 3.37 | 4.69 | 3.05 | 4.23 | 0.70 | 1.10 | 0.98 | 3.25 | 4.68 | 0.00 | 1155 |
| 洗衣机 | washer | 1 | 3 | 3.14 | 3.56 | 4.19 | 3.38 | 2.80 | 0.88 | 0.36 | 0.88 | 3.65 | 5.19 | 0.09 | 1100 |
| 手表 | watch | 1 | 2 | 2.98 | 4.17 | 4.89 | 3.14 | 3.83 | 0.93 | 0.38 | 1.00 | 3.06 | 2.54 | 0.00 | 787 |
| 瀑布 | waterfall | 3 | 2 | 2.74 | 3.72 | 4.22 | 4.38 | 2.06 | 0.98 | 0.00 | 0.98 | 3.73 | 8.27 | 0.03 | 986 |
| 喷壶 | watering can | 1 | 2 | 2.76 | 3.85 | 4.33 | 2.49 | 2.11 | 0.30 | 2.95 | 0.95 | 4.38 | 4.68 | 0.00 | 1118 |
| 水井 | well | 1 | 2 | 3.12 | 3.58 | 3.72 | 3.67 | 1.89 | 0.45 | 1.75 | 0.83 | 2.63 | 10.92 | 0.11 | 1211 |
| 鲸鱼 | whale | 5 | 2 | 3.55 | 3.84 | 3.89 | 3.00 | 2.06 | 0.38 | 1.86 | 0.75 | 3.94 | 10.30 | 0.09 | 1281 |
| 车轮 | wheel | 1 | 2 | 2.74 | 3.23 | 4.36 | 3.24 | 2.74 | 0.45 | 1.60 | 0.98 | 2.35 | 3.41 | 0.03 | 901 |
| 鞭子 | whip | 5 | 2 | 3.32 | 4.11 | 3.14 | 2.16 | 2.06 | 0.63 | 1.10 | 0.80 | 3.06 | 8.88 | 0.17 | 1303 |
| 哨子 | whistle | 1 | 2 | 2.65 | 4.51 | 4.58 | 2.08 | 2.26 | 0.58 | 1.40 | 0.98 | 2.69 | 5.74 | 0.03 | 1028 |
| 野猪 | wild boar | 2 | 2 | 2.19 | 2.41 | 2.81 | 2.81 | 1.94 | 0.30 | 1.98 | 0.65 | 4.38 | 10.55 | 0.11 | 1338 |
| 风车 | windmill | 1 | 2 | 2.74 | 3.86 | 3.94 | 4.38 | 1.84 | 0.88 | 0.52 | 0.93 | 3.94 | 10.41 | 0.06 | 1054 |
| 窗户 | window | 1 | 2 | 2.69 | 3.03 | 4.53 | 2.78 | 4.24 | 0.43 | 1.79 | 0.70 | 1.94 | 4.73 | 0.00 | 990 |
| 酒杯 | wineglass | 1 | 2 | 3.52 | 4.25 | 4.81 | 1.81 | 2.83 | 0.58 | 1.57 | 1.00 | 2.94 | 10.30 | 0.00 | 809 |
| 狼 | wolf | 1 | 1 | 2.76 | 3.29 | 4.00 | 2.38 | 1.91 | 0.65 | 1.55 | 0.68 | 2.65 | 7.45 | 0.11 | 1418 |
| 毛毛虫 | worm | 5 | 3 | 2.38 | 3.86 | 3.47 | 4.08 | 1.94 | 0.25 | 2.63 | 0.45 | 2.07 | 11.00 | 0.06 | 1162 |
| 扳手 | wrench | 1 | 2 | 2.55 | 3.36 | 4.53 | 1.51 | 2.00 | 0.45 | 1.98 | 0.78 | 5.00 | 10.93 | 0.17 | 1080 |
| 斑马 | zebra | 1 | 2 | 2.60 | 4.42 | 4.42 | 4.05 | 1.97 | 0.98 | 0.00 | 0.98 | 4.63 | 4.12 | 0.03 | 835 |
| 拉链 | zipper | 4 | 2 | 3.02 | 3.92 | 4.14 | 2.67 | 3.51 | 0.88 | 0.18 | 0.88 | 3.35 | 6.94 | 0.17 | 1140 |

Note: Name-dominant name in Chinese, the numbers in Source column: 1-Cycowicz et al. (1997), 2-Bonin et al. (2003), 3-Philadelphia Naming Test (Roach et al., 1996), 4-Zhang & Yang (2003), 5-others. VAR-image variability, IMG-image agreement, FAM-concept familiarity, VC-visual complexity, FREQ-subjective frequency, CA-concept agreement, Error- error percentage over all 41 participants, RT_harm-Harmonic means of naming latency in ms. Others are the same as in Table 2.
